# Sex and age differences in AMPK phosphorylation, mitochondrial homeostasis, and inflammation in hearts from inflammatory cardiomyopathy patients

**DOI:** 10.1111/acel.13894

**Published:** 2023-06-26

**Authors:** Maria Luisa Barcena, Greta Tonini, Natalie Haritonow, Pavelas Breiter, Hendrik Milting, Istvan Baczko, Ursula Müller‐Werdan, Yury Ladilov, Vera Regitz‐Zagrosek

**Affiliations:** ^1^ Department of Geriatrics and Medical Gerontology Charité –Universitätsmedizin Berlin, corporate member of Freie Universität Berlin, Humboldt‐Universität zu Berlin and Berlin Institute of Health Berlin Germany; ^2^ DZHK (German Centre for Cardiovascular Research) Berlin Germany; ^3^ Erich and Hanna Klessmann Institute Heart and Diabetes Centre NRW, University Hospital of the Ruhr‐University Bochum Bad Oeynhausen Germany; ^4^ Department of Pharmacology and Pharmacotherapy, Albert Szent‐Györgyi Medical School University of Szeged Szeged Hungary; ^5^ Department of Cardiovascular Surgery, Heart Center Brandenburg Brandenburg Medical School Bernau bei Berlin Germany; ^6^ Institute for Gender in Medicine, Center for Cardiovascular Research, Charité University Hospital Berlin Germany; ^7^ Department of Cardiology University Hospital Zürich, University of Zürich Zürich Switzerland

**Keywords:** acetylation, AMPK, inflammatory dilated cardiomyopathy, mitochondrial biogenesis, senescence

## Abstract

Linked to exacerbated inflammation, myocarditis is a cardiovascular disease, which may lead to dilated cardiomyopathy. Although sex and age differences in the development of chronic myocarditis have been postulated, underlying cellular mechanisms remain poorly understood. In the current study, we aimed to investigate sex and age differences in mitochondrial homeostasis, inflammation, and cellular senescence. Cardiac tissue samples from younger and older patients with inflammatory dilated cardiomyopathy (DCMI) were used. The expression of Sirt1, phosphorylated AMPK, PGC‐1α, Sirt3, acetylated SOD2, catalase, and several mitochondrial genes was analyzed to assess mitochondrial homeostasis. The expression of NF‐κB, TLR4, and interleukins was used to examine the inflammatory state in the heart. Finally, several senescence markers and telomere length were investigated. Cardiac AMPK expression and phosphorylation were significantly elevated in male DCMI patients, whereas Sirt1 expression remained unchanged in all groups investigated. AMPK upregulation was accompanied by a preserved expression of all mitochondrial proteins/genes investigated in older male DCMI patients, whereas the expression of TOM40, TIM23, and the mitochondrial oxidative phosphorylation genes was significantly reduced in older female patients. Mitochondrial homeostasis in older male patients was further supported by the reduced acetylation of mitochondrial proteins as indicated by acetylated SOD2. The inflammatory markers NF‐κB and TLR4 were downregulated in older male DCMI patients, whereas the expression of IL‐18 was increased in older female patients. This was accompanied by progressed senescence in older DCMI hearts. In conclusion, older women experience more dramatic immunometabolic disorders on the cellular level than older men.

## Graphical abstract

Older DCMI women experience more dramatic immunometabolic disorders.

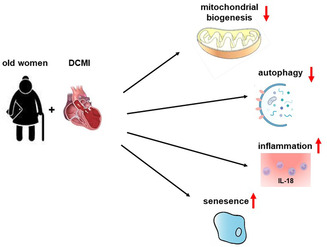

**Abbreviations**
AMPK: AMP‐activated protein kinase
BNP: brain natriuretic peptide
CK‐MB: creatinine kinase MB
CRP: C‐reactive protein
DCM: dilated cardiomyopathy
DCMI: inflammatory dilated cardiomyopathy
EF: ejection fraction
HHV6V: human herpesvirus 6
IL: interleukin
LV: left ventricular
LVAD: left ventricular assist device
NF‐κB: nuclear factor kappa‐light‐chain‐enhancer of activated B cells
PGC‐1α: peroxisome proliferator‐activated receptor‐gamma coactivator‐1 alpha
PVB19: parvovirus 19
ROS: reactive oxygen species
SASP: senescence‐associated secretory phenotype
Sirt: sirtuin
SOD2: superoxide dismutase 2
TGF‐β: transforming growth factor β
TLR4: toll‐like receptor 4
TNF‐α: tumor necrosis factor α
VEGF: vascular endothelial growth factor

## 1 INTRODUCTION

Myocarditis is characterized by myocardial inflammation (Fung et al., 2016) and is associated with immune cell infiltration, extensive scarring, and left ventricular remodeling leading to dilated cardiomyopathy (DCM), followed by heart failure and sudden death (Pollack et al., 2015). Myocarditis predominantly develops after a viral infection with, for example, parvovirus B19, human herpesvirus 6, or coxsackie B virus (Kuhl et al., 2005). Viral persistence in the heart leads to a virus‐associated inflammatory cardiomyopathy (Tschope et al., 2021), which is linked to chronic inflammation and profound cardiac remodeling due to an exacerbated activation of the immune system (Cooper Jr., 2009; Kindermann et al., 2008). In particular, macrophages and T‐cells (e.g., Th1, Th2, Th17, and FoxP3+/CD4+ T‐cells) are present during viral or toxic injury in myocarditis (Fung et al., 2016). Furthermore, the increased levels of Th1 and Th2 cytokines (e.g., interleukin (IL)‐6, IL‐1β, and tumor necrosis factor α (TNF‐α)) are closely related to the development of DCM (Fairweather et al., 2004). Our own studies and those of others have shown that sex differences exist in the inflammatory response in acute and chronic myocarditis in several animal models, which is characterized by an increased pro‐inflammatory response in male hearts (Barcena et al., 2021; Cihakova et al., 2004; Frisancho‐Kiss et al., 2006, 2007, 2009; Roberts et al., 2013). 16%–30% of patients with DCM present myocardial inflammation, suggesting a co‐occurrence of myocarditis and DCM (Blyszczuk, 2019).

Sex‐related differences in cardiac remodeling seem to be a significant part of men's increased risk of developing DCM and experiencing heart failure (Cleland et al., 2003; Luchner et al., 2002). However, in older individuals, these sex differences are less prominent, as the decrease in estrogen in older women makes them more likely to experience cardiac remodeling and cardiac dysfunction than younger women (Li & Gupte, 2017). Aging, in general, is also known to be accompanied by chronic systemic and cardiac inflammation (Barcena et al., 2022), which may further exacerbate the adverse consequences of reduced sex hormone levels in older women. In fact, the pro‐inflammatory shift in healthy older women is more prominent than in healthy male hearts (Barcena et al., 2022; Barcena de Arellano et al., 2019), while in cardiomyopathies, for example, DCM, the inflammatory pathways are also strongly activated in older male hearts (Barcena et al., 2020; Lopez‐Otin et al., 2013; Regitz‐Zagrosek & Kararigas, 2017).

The signaling pathways involved in the myocarditis‐induced remodeling leading to DCM are still poorly understood. However, increased collagen deposition and pathological fibrosis are observed during chronic inflammatory processes in myocarditis (Wynn, 2008). Due to higher testosterone levels, male hearts show elevated cardiac collagen deposition, fibrosis formation, and remodeling of the extracellular matrix in various cardiovascular diseases (Cavasin et al., 2006; Cocker et al., 2009; Coronado et al., 2012; Haddad et al., 2008), including experimental autoimmune myocarditis (EAM) (Schmerler et al., 2014).

Disturbed mitochondrial homeostasis plays a central role in many underlying cellular mechanisms involved in the development of DCM of various aetiologies (Ramaccini et al., 2020). Mitochondrial homeostasis comprises mitochondrial biogenesis and clearance control, that is, mitophagy. Both processes are important for the support of mitochondrial function, which is important for cardiomyocytes' energy balance. Two main energy sensors—AMPK and Sirt1—contribute significantly to mitochondrial homeostasis. Indeed, AMPK and Sirt1 promote PGC‐1α activity, which is a key transcription factor regulating the expression of mitochondrial proteins, and thus, mitochondrial biogenesis (Gureev et al., 2019). Furthermore, AMPK and Sirt1 also control cellular autophagy activity, and thus, mitophagy (Jang et al., 2018; Sacitharan et al., 2020), a key process in the elimination of dysfunctional or damaged mitochondria.

Aging is accompanied by a strong downregulation of AMPK and Sirt1 activity (Barcena de Arellano et al., 2019; PLOS ONE Editors, 2022) which leads to impaired mitophagy, an accumulation of dysfunctional mitochondria, and ROS formation and which may trigger inflammation and further exacerbate post‐myocarditis remodeling. It is worth noting that the release of mitochondrial DNA from damaged mitochondria into the cytosol may lead to an inflammatory response via Toll‐like receptors and STING‐dependent inflammasome activation (Riley & Tait, 2020). Furthermore, AMPK and Sirt1 suppress NF‐κB signaling both directly and indirectly, which can subsequently reduce the expression of pro‐inflammatory factors (Salminen et al., 2011; Yeung et al., 2004). Our recent report (Barcena de Arellano et al., 2019) demonstrated the association between the age‐related reduction in AMPK activity and inflammation in the human heart.

Aside from Sirt1, the downregulation of another key cellular sirtuin, mitochondrial Sirt3, was also observed in aging hearts (Zhang et al., 2021). Sirt3 is a key mitochondrial deacetylase supporting mitochondrial dynamics and function via the deacetylation of numerous mitochondrial enzymes, including superoxide dismutase 2 (SOD2), whose activity is suppressed by hyperacetylation (Xu et al., 2020). Thus, the reduced expression of Sirt3 is accompanied by reduced ATP synthesis and elevated ROS formation.

In the present study, we aimed to investigate sex‐ and age‐related effects on mitochondrial homeostasis, autophagy, inflammation, and senescence in heart biopsies of patients with inflammatory dilated cardiomyopathy (DCMI). We also analyzed the relevant energy‐sensing pathways, that is, AMPK and Sirt1. The study documents a reduced expression of mitochondrial proteins and mitochondria‐encoded genes in older female patients, which was accompanied by a pro‐inflammatory shift. Another interesting finding is that the acetylation rate of mitochondrial proteins, for example, the acetylation of SOD2, was significantly lower in the myocardium of older male than older female cardiomyopathy patients, suggesting that mitochondrial homeostasis is preserved in older male patients.

## 2 MATERIALS AND METHODS

### 2.1 Human left ventricular DCMI samples

Human lateral left ventricular (LV) wall or left ventricular apex tissue samples from patients with DCMI caused by viral myocarditis was retrospectively collected during organ transplantation or left ventricular assist device (LVAD) (men = 10–20 and women = 10–15). At the time of sample collection, 61% of the patients underwent organ transplantation, while a LVAD was implanted in 39% of the patients. The tissue was frozen immediately after collection in liquid nitrogen and stored at −80°C. The donors had an ejection fraction (EF) of <35% (Table 1) and were between 23 and 70 years of age. We divided the DCMI samples into four groups: younger (23–40 years; male: *n* = 5–10 and female: *n* = 3–5) and older (50–70 years; male: *n* = 5–10 and female: *n* = 5–10) individuals.

**TABLE 1:** Characterization of patients with myocarditis‐related cardiomyopathy.

	Men		Women	
	Young	Old	Young	Old
Age	28.50 ± 6.14	60.88 ± 7.22	34.66 ± 7.57	62.40 ± 3.21
BMI	25.61 ± 4.52	25.72 ± 2.03	30.16 ± 1.32	27.39 ± 1.59
EF (%)	21.87 ± 2.72^a^	22.66 ± 1.12^b^	30.85 ± 3.12	32.50 ± 3.68
FS (%)	19.37 ± 5.18	15.00 ± 1.81	18.85 ± 2.71	21.08 ± 4.00
BNP (pg/mL)	1407 ± 615	401 ± 100	228 ± 42	596 ± 145
Troponin I (pg/mL)	41.43 ± 9.75	43.90 ± 11.96^b^	21.66 ± 5.11	13.89 ± 3.13
CRP (mg/dL)	0.86 ± 6.21	1.05 ± 0.28^b^	2.13 ± 1.47	0.38 ± 0.07
CK‐MB (ng/dL)	9.05 ± 2.55	1.83 ± 0.28	2.99 ± 1.06	41.63 ± 23.69
CD45 (cardiac tissue)	1.05 ± 0.15a	0.81 ± 0.24	0.36 ± 0.02^c^	1.39 ± 0.61
CD11b (cardiac tissue)	1.16 ± 0.20	0.89 ± 0.15	0.49 ± 0.03	0.89 ± 0.19
PVB19 positive	33%	61%	100%	63%
HHV6B positive	22%	15%	0%	18%
PVB19 and HHV6B positive	44%	23%	0%	18%
Number of virus copies	1323 ± 565	904 ± 428	1189 ± 206	732 ± 522

*Note*: Data are shown as the means ± SEM. *n* = 3–12. Echocardiography analyses were performed to analyze EF, FS, and PW‐ED. Elisa analyses were performed to analyzed BNP, Troponin I, CRP, and CK‐MB. qRT‐PCR analyses were performed to analyze CD45 and CD11b expression. Viral infection was proved using PCR analysis.

Abbreviations: BMI, body mass index; BNP, brain natriuretic peptide; CK‐MB, creatine kinase muscle‐brain type; CRP, C‐reactive protein; EF, ejection fraction; FS, fractional shorting; LV‐PW, left ventricle posterior wall thickness; PVB19, parvovirus b19; HHV6B, human herpesvirus 6.

^a^ *p* < 0.05 for men versus women (young).

^b^ *p* < 0.05 for men versus women (old).

^c^ *p* < 0.05 for young versus old (women).

We obtained informed consent from all donors. Sample collection and the experimental protocols were approved by the Scientific Board at the Heart and Diabetes Center (HDZ) NRW (21/2013) and at the Charité – Universitätsmedizin Berlin (EA2/158/16). All experiments with the samples were performed in accordance with German regulations and the ethical standards as laid down in the Declaration of Helsinki.

### 2.2 Human left ventricular Non‐Diseased samples

Human non‐diseased lateral left ventricular (LV) wall tissue was retrospectively collected from organ donors (men = 19 and women = 19). The donors were between 17 and 68 years of age. The LV samples were divided into four groups: younger (17–40 years; male: *n* = 9 and female: *n* = 9) and older (50–68 years; male: *n* = 10 and female: *n* = 10) individuals. The hearts were immediately put on ice in a Custodiol® solution (15 mmoL/L sodium chloride, 9 mmoL/L potassium chloride, 1 mmoL/L potassium hydrogen 2‐ketoglutarate, 4 mmoL/L magnesium chloride 6 H_2_O, 18 mmoL/L histidine HCl H_2_O, 180 mmoL/L histidine, 2 mmoL/L tryptophan, 30 mmoL/L mannitol, 30 mmoL/L calcium chloride 2 H_2_O, 50 mEG Anion: Cl‐, 300 mosmol/kg osmolality) for cardioplegia and multi‐organ protection until dissection. After dissection, the samples were immediately frozen in liquid nitrogen and stored at −80°C.

The donors were healthy individuals. The cause of death was head or brain injury due to accidents (e.g., basilar skull fracture or brain contusion). The organs of the donors were used for multi‐transplantation, but the hearts could not be transplanted for logistical reasons.

Informed consent from all donors or their legal guardians was obtained. The Scientific Board at the Hungarian Ministry of Health (ETT‐TUKEB: 4991–0/2010‐1018EKU) approved the sample collection and the experimental protocols. All research was performed in accordance with the German and Hungarian regulatory guidelines and the ethical standards as laid down in the Declaration of Helsinki.

### 2.3 Measurement of clinical parameters

Cardiac function (ejection fraction and fractional shortening) was analyzed via echocardiography at the Heart and Diabetes Center (HDZ) NRW up to 3 months before LVAD implantation or heart transplantation. In addition, standard biochemical parameters, for example, brain natriuretic peptide (BNP), troponin I, creatinine kinase MB (CK‐MB), and C‐reactive protein (CRP), were routinely measured in the clinical laboratory at the HDZ NRW up to 3 months before operation.

### 2.4 RNA extraction and quantitative Real‐Time PCR

Both total RNA isolation from cardiac human tissue as well as a quantitative real‐time PCR were performed as previously described (Barcena et al., 2020). The mRNA contents of target genes were normalized to the expression of hypoxanthine phosphoribosyl transferase (HPRT) and ribosomal protein lateral stalk subunit P0 (RPLP0). The purity of the isolated RNA was analyzed with the Caliper LabChip bioanalyzer (Agilent Technologies).

### 2.5 Protein extraction and immunoblotting

Cardiac samples from both DCMI and non‐diseased hearts were homogenized in Laemmli buffer (253 mM Tris/HCL pH 6.8, 8% SDS, 40% glycerin, 200 mM DDT, 0.4% bromophenol blue). Proteins were quantified using the BCA Assay (Thermo Scientific Pierce Protein Biology). Equal amounts of total proteins were separated on SDS‐polyacrylamide gels and transferred to a nitrocellulose membrane. The membranes were immunoblotted overnight with the following primary antibodies: Sirt1 (1:1000, #8469, Cell Signaling), AMPK (1:2000, #2532L, Cell Signaling), p‐AMPK (1:2000, Thr172, #2535L, Cell Signaling), PGC‐1α (1:1000, #54481, Abcam), TOM40 (1:1000, #sc365466, Santa Cruz), TIM23 (1:5000, #611222, BD), Sirt3 (1:1000, #5490, Cell Signaling), SOD2 (1:1000, #13194, Cell Signaling), ac‐SOD2 (1:1000, #ab13533,Abcam), catalase (1:1000, #14097S, Cell Signaling), ATG5 (1:1000, #12994, Cell Signaling), p62/SQSTM1 (1:1000, #MAB8028, R&D systems), LC3 (1:1000, #4599, Cell Signaling), LAMP2 (1:1000; #NB300‐591, Novus), NFκBp65 (1:200, #sc‐8008, Santa Cruz), lamin B1 (1:1000, #13435, Cell Signaling), p53 (1:500, #sc‐6243, Santa Cruz), MMP3 (1:1000, #14351, Cell Signaling) and phospho‐histone‐H2A.X (Ser139) (1:1000, #9718, Cell Signaling). Equal sample loading was confirmed by an analysis of GAPDH (1:1000, #8469, Cell Signaling), actin (1:1000, #sc1616‐R, Santa Cruz), or HSP60 (1:1000, #4870, Cell Signaling). Immunoreactive proteins were detected using ECL Plus (GE Healthcare) and quantified with ImageLab (version 5.2.1 build 11, Bio‐Rad Laboratories (USA)). Original blots are shown in a supplemental file.

### 2.6 Mitochondrial mass analysis

To analyze the amounts of nuclear and mitochondrial DNA (mt‐DNA), a quantitative real‐time PCR was performed. The measurement of mitochondrial content was conducted with the ratio of mt‐DNA to nuclear DNA (Jayarajan et al., 2019). Mt‐DNA‐specific mt‐RNR2 primers (forward 5'‐CCACATCTGCCGAGACGTAA‐3' and reverse 5'‐TAGTCCTCGTCCCACATGGA‐3') and nuclear DNA‐specific β‐globin primers (forward 5'‐AAGTACCACTAAGCCCCCTTTC‐3' and reverse 5'‐GGGAACACAAAAGACCTCTTCTGG‐3') for SYBR Green were used.

### 2.7 Telomere length measurement

Telomere length was measured in cardiac samples from both DCMI and non‐diseased hearts using the Absolute Human Telomere Length quantification qPCR assay kit (ScienCell Research Laboratories) according to the manufacturer's protocol via quantitative real‐time PCR (O'Callaghan & Fenech, 2011).

### 2.8 Masson's trichrome staining

5 μm cryosections of the human left ventricle were stained with Masson's trichrome (Dako, Germany), according to the manufacturer's instructions to quantify fibrotic tissue (magnification ×200). Images were acquired with the Axiophot microscope (Zeiss). The overall fibrosis was determined via semiquantitative, visual evaluation. All sections were blindly evaluated by three different investigators.

### 2.9 Immunofluorescence

5 μm cryosections of the human left ventricle were stained with antibodies against IL‐18 (1:500, PA5‐79479, ThermoFisher Scientific). The secondary antibody anti‐rabbit FITC (1:100) (Dianova) was applied according to the manufacturer's protocol. Nuclei were stained using DAPI (1:50000) (Sigma) and cells were mounted with Fluoromount G (Southern Biotech). Negative controls were performed by omitting the primary antibodies. Images were acquired using a Leica TCS SPE II confocal unit with Leica DMI 6000 microscope (Leica Microsystems GmbH). All evaluations were performed in a blinded manner.

### 2.10 Statistical analysis

The data are given as the means ± SEM. The GraphPad Prism 7 (GraphPad Software) was used for the statistical analysis. The data were evaluated using the non‐parametric test (Mann–Whitney test, for two independent groups). Statistical significance was accepted when *p* < 0.05.

## 3 RESULTS

### 3.1 Characterization of patients with DCMI

To characterize the patients with DCMI, body mass index (BMI), cardiac functional parameters, and inflammatory state were analyzed. No differences in BMI between the groups were observed (*p* > 0.05) (Table 1). In 68% of the samples, parvovirus B19 (PVB19) was detected. 18% of the samples were positive for human herpesvirus 6B (HHV6B) and 14% for both PVB19 and HHV6B (Table 1). In 70% of the cardiac biopsies, a virus replication between 100 and 3799 copies was detected, while 30% of the cardiac tissue had fewer than 100 virus copies (Table 1). Interestingly, patients infected only with HHV6B had fewer virus copies than the other groups (data not shown). Of note, younger patients with DCMI had a higher number of virus copies in comparison to older patients in a sex‐independent manner; however, the difference was not significant (*p* > 0.05) (data not shown). Regarding medication, 36% of the patients were treated with ACE inhibitors, 22% with an AT1‐R blocker, and 69% with a beta blocker. In addition, 81% of the patients with DCMI took diuretics.

In patients with DCMI, cardiac function (ejection fraction and fractional shortening) as well as cardiac parameters (BNP, troponin I, CK‐MB, and CRP) were analyzed (Table 1). The ejection fraction was significantly lower in men compared to women in an age‐independent manner (*p* < 0.05). Furthermore, troponin I and CRP were significantly increased in older men in comparison with older women with DCMI (*p* < 0.05) (Table 1).

Pathological characterization of the cardiac tissue from patients with DCMI revealed immune cell infiltrates in a sex‐ and age‐independent manner (data not shown). Interestingly, CD45 mRNA was significantly lower in the myocardium of younger women with DCMI when compared to older women and younger men (*p* < 0.05) (Table 1), while the mRNA values of cardiac CD11b did not differ between the groups (*p* > 0.05) (Table 1). In addition, cardiac biopsies from patients with DCMI showed increased pathological fibrosis formation in younger and older men and older women, but not in younger women (Figure S1).

### 3.2 AMPK and pAMPK expression elevated in male patients with DCMI

**FIGURE 1:**
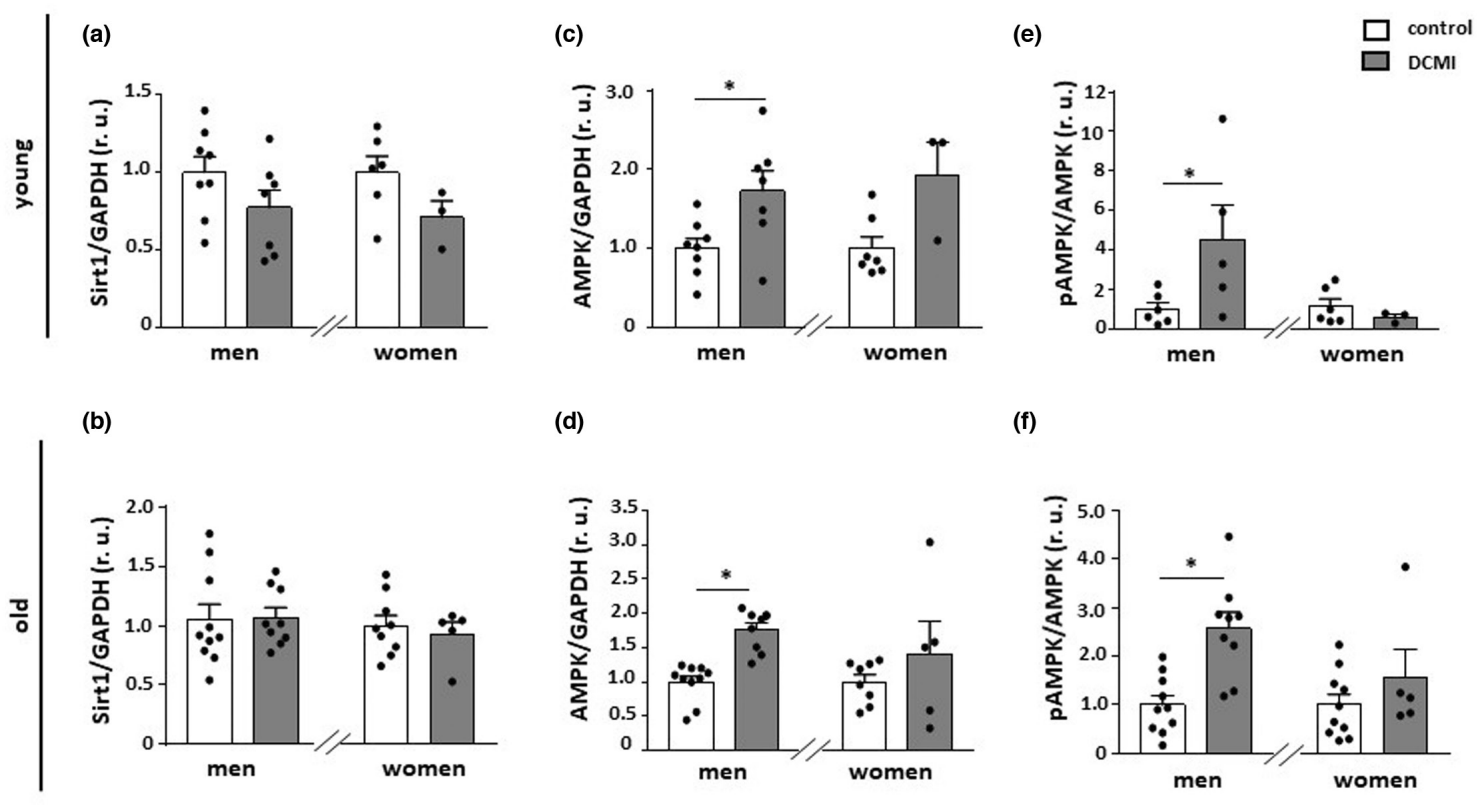
Alterations in the expression of Sirt1 and AMPK. Statistics from western blot analysis of (a and b) Sirt1, (c and d) total AMPK, and (e and f) phosphorylated AMPK (Thr172) performed with lysates of cardiac tissue from diseased and non‐diseased, younger, and older men and women. Data are shown as the means ± SEM (*n* = 3‐10/group). Mann–Whitney Test; *p < 0.05 versus corresponding control. All data were normalized to the corresponding control and expressed in relative units (r.u.).

Sirt1 and AMPK play a key role in metabolic regulation in the heart and their expression is downregulated in aged myocardium (Barcena et al., 2022; Barcena de Arellano et al., 2019). However, the existence of any age‐ and sex‐related differences in DCMI has yet to be determined. In the current study, DCMI did not affect Sirt1 expression (*p* > 0.05), whereas a marked upregulation of AMPK was observed in male individuals with DCMI in comparison with non‐diseased male control in an age‐independent manner (*p* < 0.05) (Figure 1a–d). Furthermore, the phosphorylation of AMPK, an indicator of AMPK activity, was also significantly upregulated in younger and older men with DCMI in comparison with non‐diseased male control (*p* < 0.05) (Figure 1e,f). No alterations in the expression of AMPK or pAMPK were found in women with DCMI when compared to a non‐diseased control group (*p* > 0.05) (Figure 1c–f).

### 3.3 Expression of mitochondrial proteins is reduced in older women with DCMI

**FIGURE 2:**
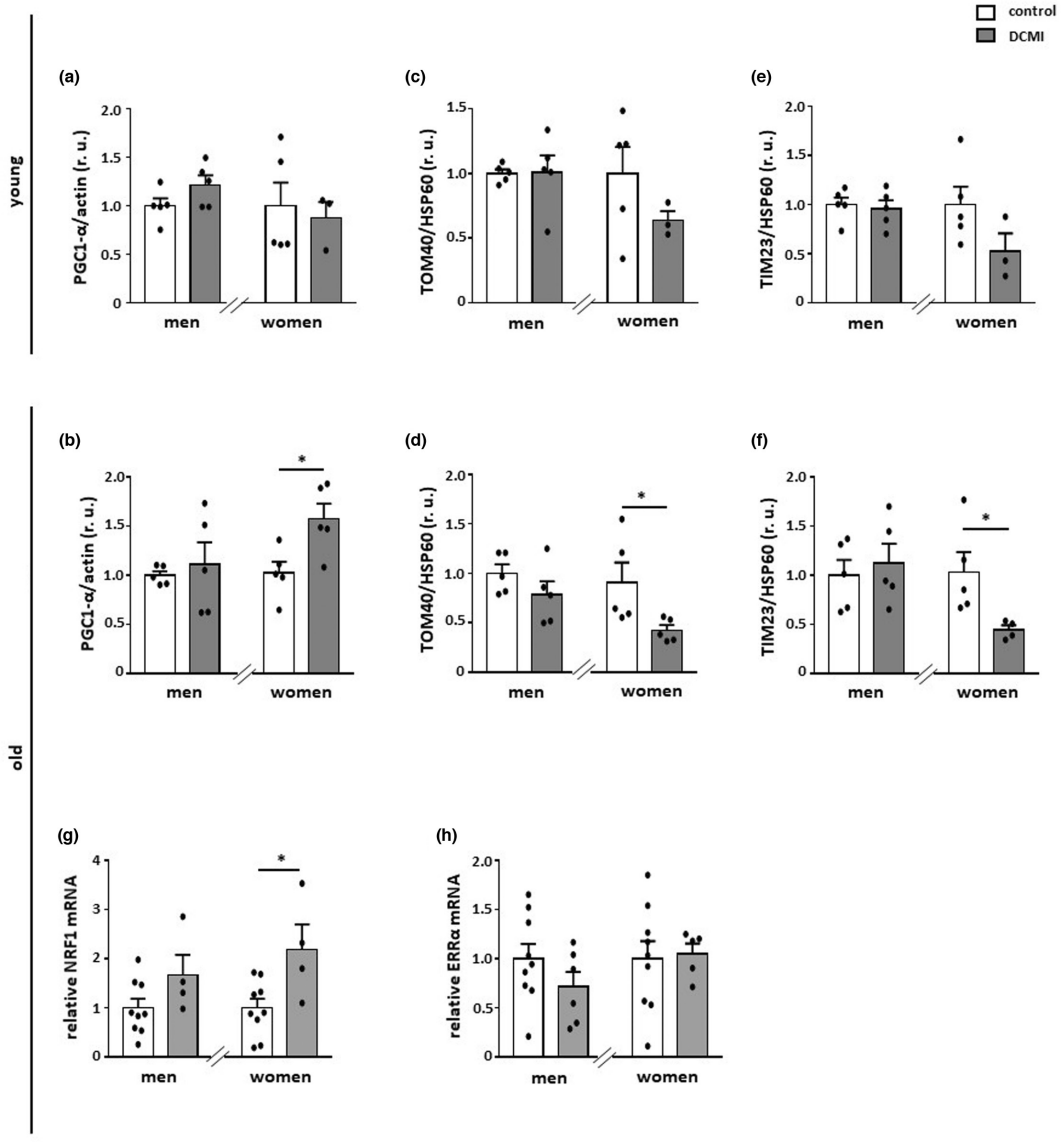
Alterations in the expression of PGC‐1*α* and mitochondrial proteins. Statistics from western blot expression analysis of (a and b) PGC‐1*α*, (c and d) TOM40, and (e and f) TIM23, performed with lysates of cardiac tissue from diseased and non‐diseased, younger and older men and women. (g and h) Relative mRNA expression of NRF1 and ERRα in diseased and non‐diseased cardiac tissue in older men and women. Data are shown as the means ± SEM (*n* = 3‐9/group). Mann–Whitney Test; *p < 0.05 versus corresponding control. All data were normalized to the corresponding control and expressed in relative units (r.u.).

**FIGURE 3:**
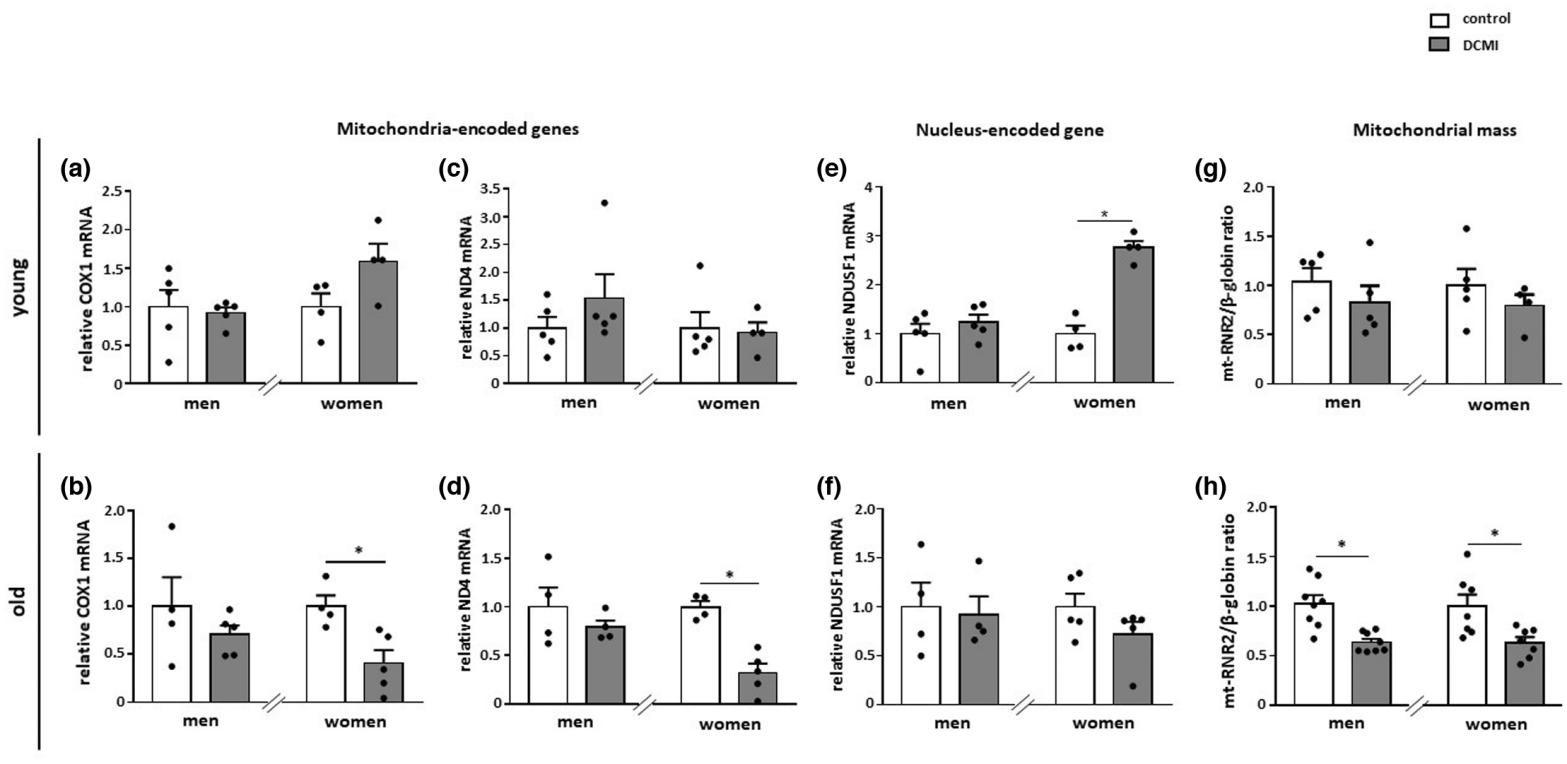
Alterations in the expression of mitochondrial genes and mitochondrial mass. (a–f) Relative mRNA expression of mitochondria‐ and nucleus‐encoded genes in diseased and non‐diseased cardiac tissue in younger and older men and women. (g‐h) Mitochondrial mass was analyzed as the mt‐RNR2/β‐globin ratio. Data are shown as the means ± SEM (*n* = 3‐8/group). Mann–Whitney Test; *p < 0.05 versus corresponding control. All data were normalized to the corresponding control.

As AMPK upregulation may promote mitochondrial biogenesis, we examined whether DCMI affects the expression of mitochondrial proteins and genes. Applying western blot or PCR assays, we found a downregulation of key mitochondrial import machinery proteins (Tom40 and Tim23) and oxidative phosphorylation (OXPHOS) genes (*cox1* and *nd4*) in older women with DCMI when compared to the non‐diseased control (*p* < 0.05) (Figure 2d,f, Figure 3b,d). Surprisingly, the expression of PGC‐1*α*, a key transcription factor controlling the expression of mitochondrial genes, was rather elevated in this patient group (*p* < 0.05) (Figure 2b). These myocarditis effects observed in women were not detected in older male DCMI hearts (*p* > 0.05). Consistent with the increased PGC‐1*α* expression in older women with DCMI, mRNA level of NFR1, a downstream transcription factor of PGC‐1*α*, in this group was also significantly upregulated (*p* < 0.05) (Figure 2g). The expression of other PGC‐1*α* target ERRα was not affected in the hearts of older patients with DCMI (*p* > 0.05) (Figure 2h). Thus, DCMI is accompanied by a reduced expression of mitochondrial proteins and genes specifically in older female patients.

**FIGURE 4:**
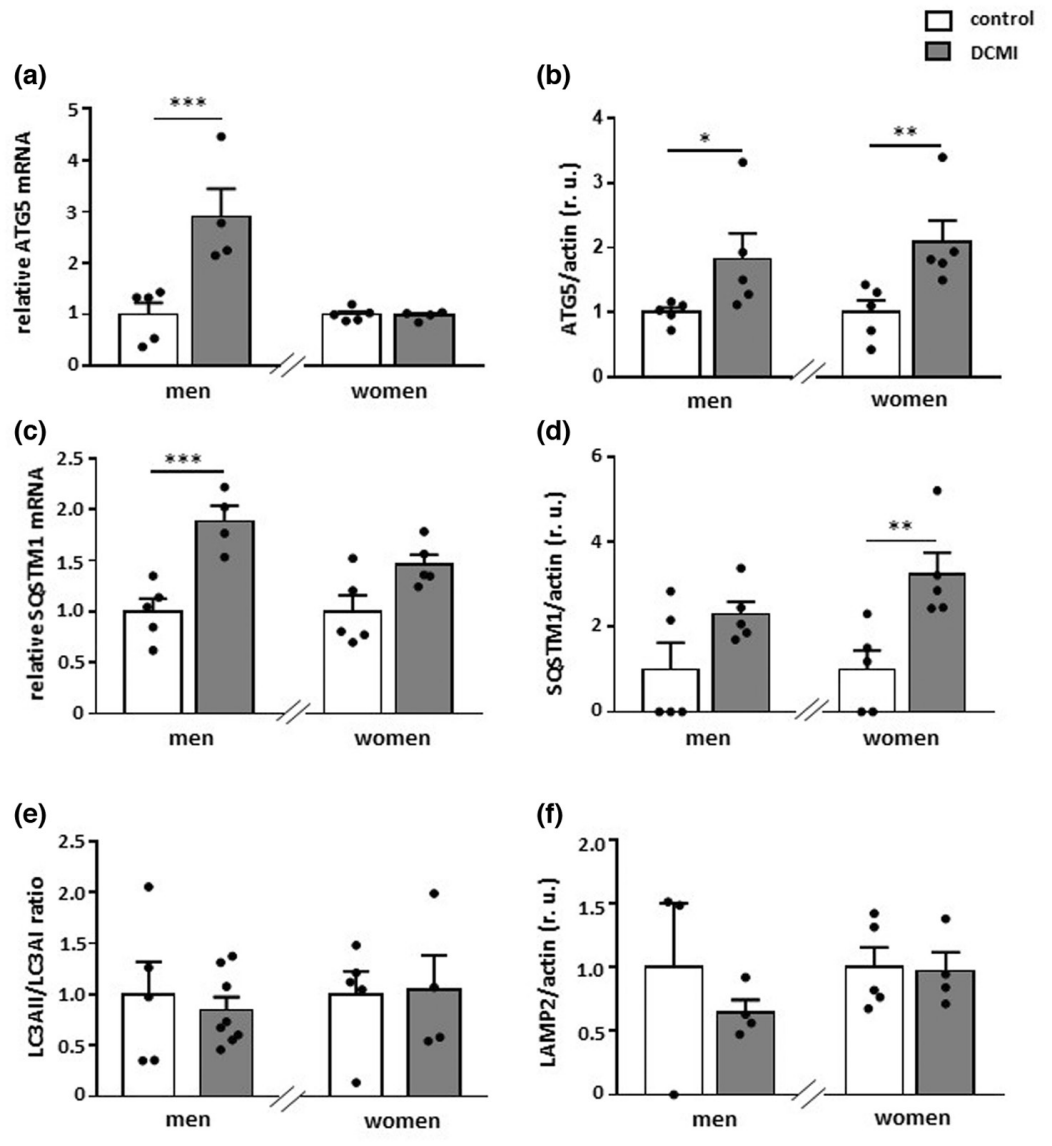
Alterations in the expression of autophagy markers. Statistics from expression analyses of (a and b) ATG5, (c and d) SQSTM1 at the mRNA and protein levels, (e) LC3II/LC3I ratio, and (f) LAMP2 performed with lysates of cardiac tissue from diseased and non‐diseased older men and women. Data are shown as the means ± SEM (*n* = 3‐8/group). Mann–Whitney Test; *p < 0.05, **p < 0.01, ***p < 0.001 versus corresponding control. All data were normalized to the corresponding control and expressed in relative units (r.u.).

We further examined whether the reduced expression of the mitochondrial proteins may affect mitochondrial mass in DCMI. Analysis of mitochondrial mass—measured as a ratio of mitochondrial DNA/nuclear DNA—revealed, as expected, a significant reduction of mitochondrial mass in older, but not younger, female patients (*p* < 0.05 and *p* > 0.05, respectively) (Figure 3g,h). Surprisingly, a similar significant reduction of mitochondrial mass was also observed in older male patients (*p* < 0.05). We hypothesized that, in this patient group, mitochondrial turnover may be accelerated due to selective autophagy. When examining typical autophagy markers, we found an upregulation of ATG5 and SQSTM1 at the mRNA level in older male patients (*p* < 0.01) (Figure 4a–d), which indicates enhanced autophagy. Of note, ATG5 and SQSTM1 were significantly upregulated in older women at the protein level (*p* < 0.01), while the transcription of these proteins was unchanged (*p* > 0.05), suggesting that the autophagy was disrupted in older female patients (Figure 4). In contrast, the LC3II/LC3I ratio was not altered in patients with DCMI (*p* > 0.05) (Figure 4e). Similarly, the lysosomal membrane protein LAMP2 was not altered in DCMI (*p* > 0.05) (Figure 4f).

### 3.4 DCMI affects mitochondrial protein acetylation in an age‐ and sex‐dependent manner

**FIGURE 5:**
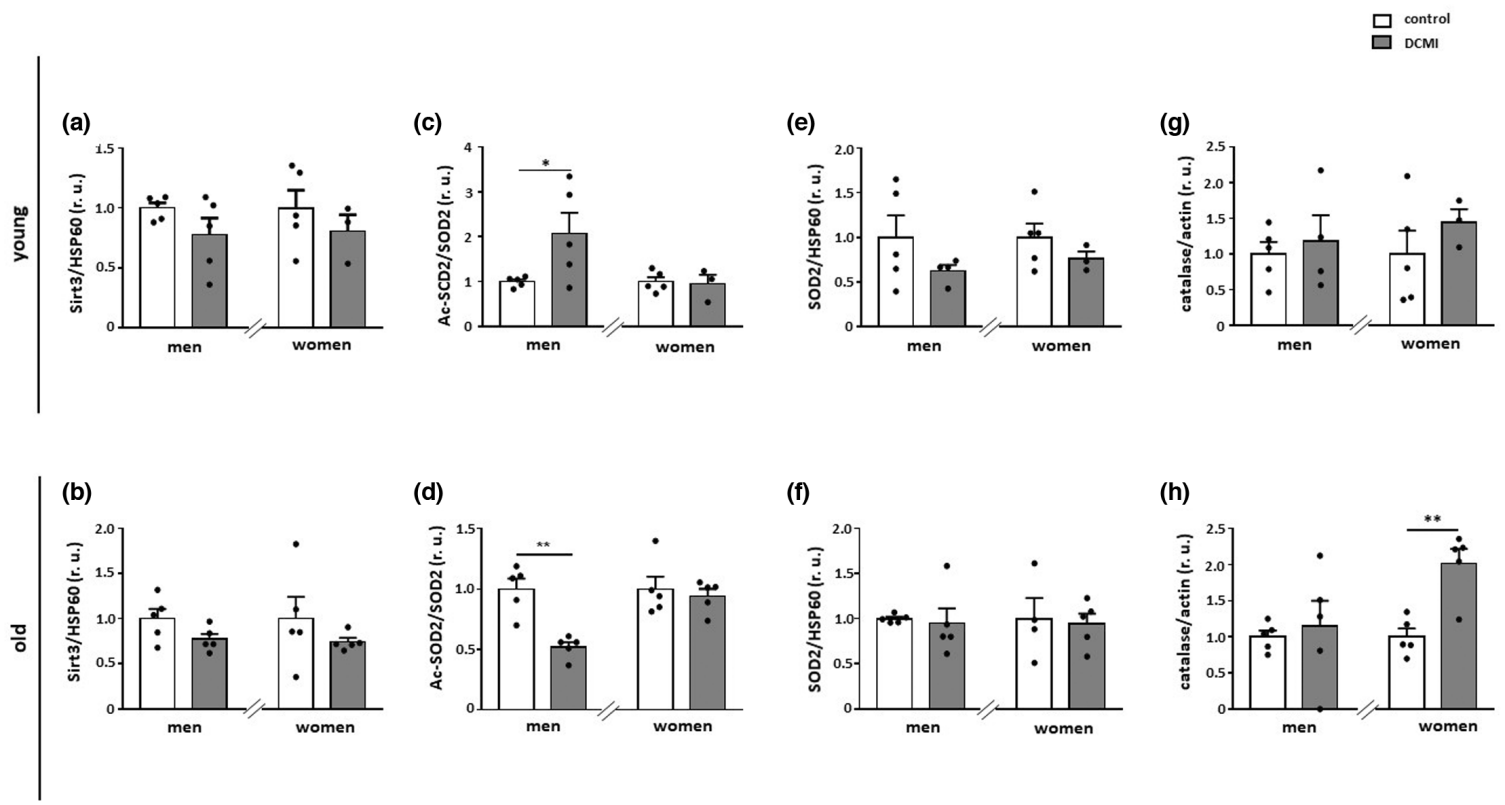
Alterations in mitochondrial acetylation state and expression of anti‐oxidative enzymes. Statistics from western blot analyses of (a and b) Sirt3, (c and d) acetylated SOD2 (acSOD2), (e and f) total SOD2, and (g and h) catalase performed with lysates of cardiac tissue from diseased and non‐diseased, younger and older men and women. Data are shown as the means ± SEM (*n* = 3‐5/group). Mann–Whitney Test; *p < 0.05, **p < 0.01 versus corresponding control. All data were normalized to the corresponding control and expressed in relative units (r.u.).

Acetylation is a key regulator of the enzymatic activity of many proteins. In mitochondria, lysine acetylation is controlled by the deacetylase Sirt3, which is the only mitochondrial sirtuin with robust deacetylation activity (Onyango et al., 2002). In the present study, no effects of DCMI were found on Sirt3 expression in all groups investigated (*p* > 0.05) (Figure 5a,b).

To further investigate the acetylation of mitochondrial proteins, western blot analysis of acetylated SOD2, a widely used marker of mitochondrial protein acetylation state (Qiu et al., 2010), was applied. The acetylation of SOD2 was significantly reduced in older male patients with DCMI (*p* < 0.01), whereas it was increased in younger men (*p* < 0.05) (Figure 5c,d). No changes in total SOD2 expression were found in all groups investigated (*p* > 0.05) (Figure 5e,f).

To further investigate alterations in the antioxidant machinery, we analyzed the expression of catalase which, in addition to the SOD2, is a prominent cellular anti‐oxidative enzyme located predominantly in peroxisomes (Karnati et al., 2013). In the present study, DCMI only significantly promoted the expression of catalase in older women (*p* < 0.01) (Figure 5h).

### 3.5 Sex differences in the pro‐inflammatory state in patients with DCMI

**FIGURE 6:**
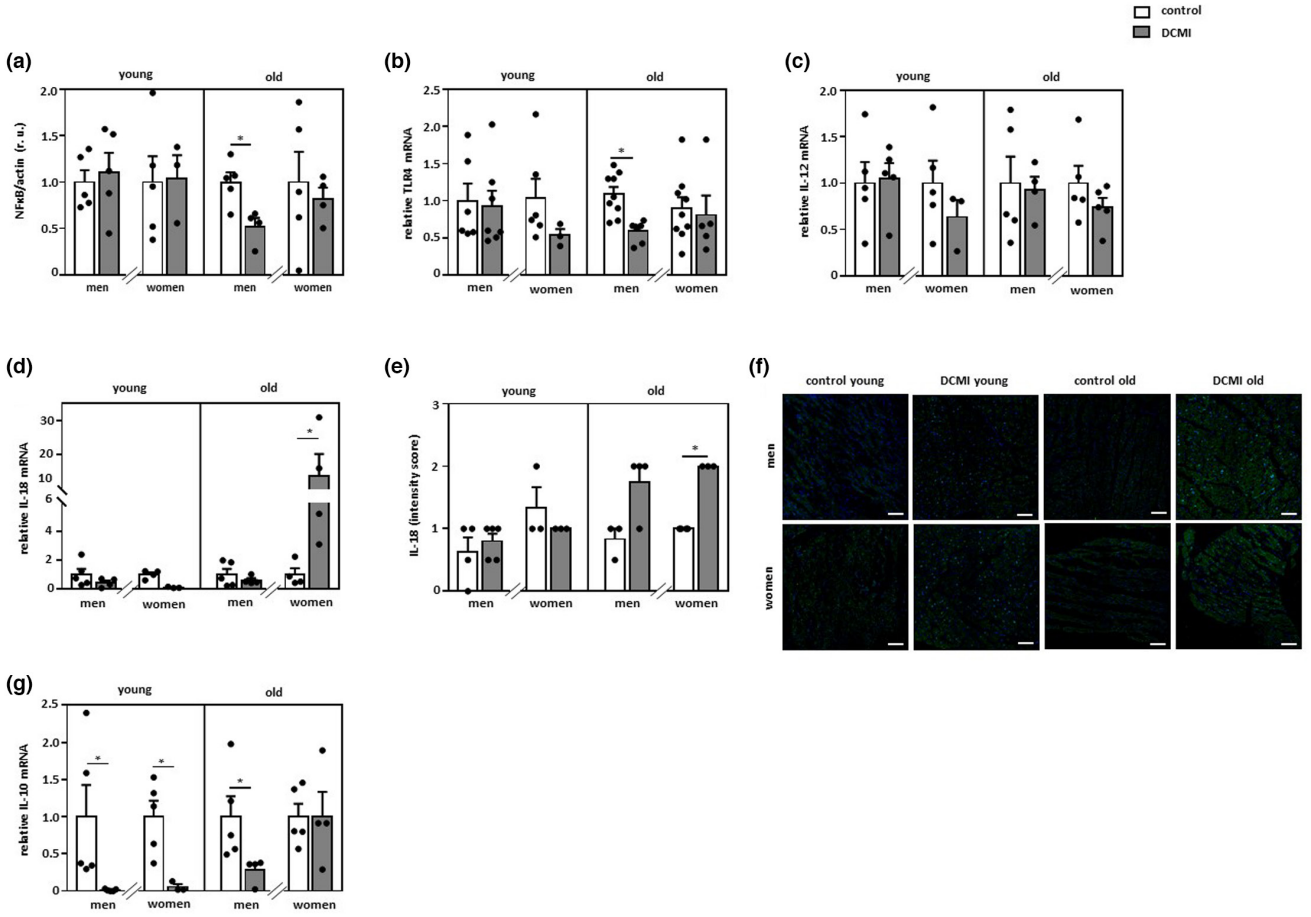
Alterations in pro‐ and anti‐inflammatory markers. Statistics from western blot analyses of (a) NF‐κB and real‐time PCR analyses of (b) TLR4, (c) IL‐12, (d) IL‐18, and (g) IL‐10 mRNA expression performed with lysates of cardiac tissue from diseased and non‐diseased, younger and older men and women. Data are shown as the means ± SEM (*n* = 3‐9/group). All data were normalized to the corresponding control and western blot data expressed in relative units (r.u.). (e and f) Representative images and statistics of IL‐18 staining of cardiac tissue from younger and older men and women with DCMI (*n* = 3‐5/group). Magnification 200×. Scale bar = 100 μm. Mann–Whitney Test; *p < 0.05 versus corresponding control.

We have previously reported that the expression of pro‐inflammatory mediators is promoted during aging processes in non‐diseased human hearts (Barcena de Arellano et al., 2019) and in older patients with DCM (Barcena et al., 2020). Thus, we examined whether the expression of pro‐inflammatory mediators is affected in DCMI. NFκB, a key regulator of cytokine expression, was significantly downregulated in older male DCMI patients (*p* < 0.05), while it did not change in female hearts (*p* > 0.05) (Figure 6a). Similarly, cardiac TLR4 mRNA expression was significantly decreased specifically in older male patients with DCMI (*p* < 0.05), (Figure 6b). The expression of the pro‐inflammatory IL‐12 changed in neither younger nor older individuals with DCMI in comparison to the corresponding controls (*p* > 0.05) (Figure 6c).

The mRNA level of the pro‐inflammatory cytokine IL‐18 was only dramatically increased in older female patients (*p* < 0.05) (Figure 6d), while immunofluorescence staining revealed an elevation of IL‐18 in older DCMI patients, which was significantly increased only in older female DCMI patients (*p* < 0.05) (Figure 6e,f). Analysis of the anti‐inflammatory marker IL‐10 expression revealed its significant reduction in younger DCMI hearts in a sex‐independent manner (*p* < 0.05), whereas it was significantly decreased only in the male hearts among older individuals with DCMI (*p* < 0.05) (Figure 6g).

### 3.6 DCMI induces cellular senescence

**FIGURE 7:**
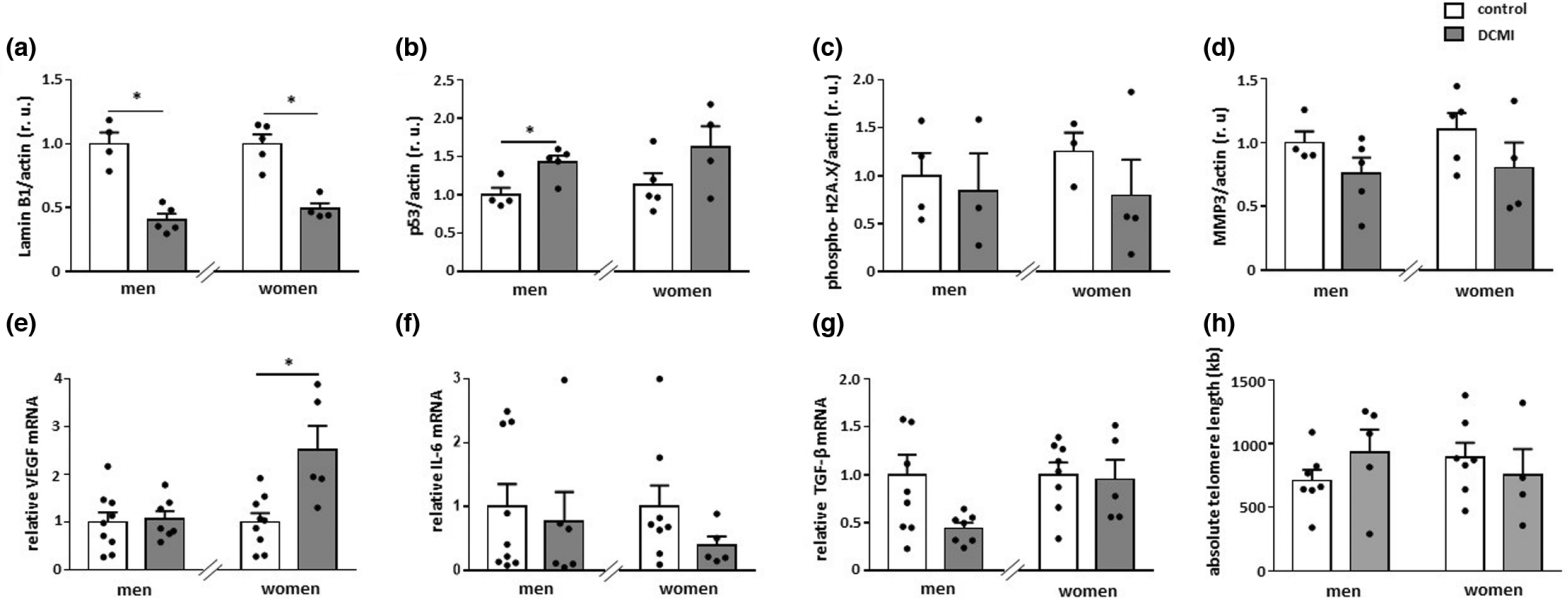
Alterations in the expression of senescence markers. Statistics from western blot analyses of (a) lamin B1, (b) p53, (c) phospho‐H2A.X, and (d) MMP3 and real‐time PCR analyses of (e) VEGF, (f) IL‐6, and (g) TGF‐β mRNA expression performed with lysates of cardiac tissue from diseased and non‐diseased, older men and women. Data are shown as the means ± SEM (*n* = 3‐9/group). Mann–Whitney Test; *p < 0.05 versus corresponding control. All data were normalized to the corresponding control and expressed in relative units (r.u.). (H) Absolute telomere length measurement performed with DNA of cardiac tissue from diseased and non‐diseased, older men and women. Data are shown as the means ± SEM (*n* = 4‐7/group).

Chronic inflammation can promote cellular senescence (Barcena et al., 2022). As we observed an inflammatory impairment in the hearts of patients with DCMI, senescence markers were investigated in this group. Loss of nuclear protein lamin B1, which is involved in DNA repair and chromatin remodeling (Shimi et al., 2011), is an important biomarker of cellular senescence (Freund et al., 2012). The expression of nuclear lamin B1 in our study was significantly downregulated in the heart of older (Figure 7a), but not younger patients with DCMI (data not shown) in a sex‐independent manner. Moreover, p53 was increased in older hearts of male and female DCMI patients (*p* < 0.05 and *p* > 0.05, respectively) (Figure 7b). In contrast, cardiac phospho‐H2A.X and MMP3 expression was not affected in DCMI patients (*p* > 0.05) (Figure 7c,d and data not shown). At the mRNA level, we found a significant upregulation of VEGF in the hearts of older DCMI women (*p* < 0.05), while in older men it remained unchanged (*p* > 0.05) (Figure 7e). The senescence markers IL‐6 and TGF‐β were not affected by DCMI in the hearts of older men and women (*p* > 0.05) (Figure 7f,g). Finally, the analysis of the absolute telomere length revealed no effects of DCMI either in younger (data not shown) or in older patients (Figure 7h).

## 4 DISCUSSION

In this study, we investigated the sex‐ and age‐dependent effects of DCMI on cardiac markers of metabolism, mitochondrial homeostasis, autophagy, inflammation, and senescence. The main findings of this work are as follows: (1) The expression of total AMPK and pAMPK was elevated in male DCMI patients independent of age. (2) The expression of mitochondrial proteins was markedly downregulated in older female patients, while it remained stable in male patients. (3) This was accompanied by reduced acetylation of the mitochondrial matrix proteins, assessed by SOD2 acetylation, in older men with DCMI and elevated acetylation in younger men with DCMI. (4) Hearts from older female patients with DCMI showed a pro‐inflammatory shift. (5) DCMI promoted cellular senescence in older patients.

Previous reports have emphasized the significant impact of disturbed metabolic homeostasis on the progress of cardiomyopathy of various origins (Asakura & Kitakaze, 2009; Fang et al., 2020; Jefferies & Towbin, 2010; Varga et al., 2015) In the present study, we used a cohort of patients in the end stage of DCMI. We first examined the expression of two main metabolic regulators in mammalian cells: Sirt1 and AMPK. Though cardiomyopathy did not affect Sirt1 expression, a significant upregulation of total AMPK was found in both older and younger male patients. This effect was accompanied by a marked upregulation of the phosphorylated form of AMPK, which is its active form. As AMPK is an important regulator of mitochondrial homeostasis, for example, biogenesis and clearance, we suspected such a pronounced upregulation of AMPK might affect mitochondrial biology.

Analyzing the expression of mitochondrial proteins (TOM40 and TIM23), as well as the expression of mitochondrial genes (*cox1* and *nd4*), we found no significant effects of cardiomyopathy in younger or older male patients. It is worth noting that in older, but not younger, female patients, cardiomyopathy led to a significant downregulation of all these mitochondrial markers, although the expression of TOM40 and TIM23 was not significantly but notably reduced in younger female patients. These data argue for a disturbance of mitochondrial biogenesis specifically in older female patients.

The downregulation of mitochondrial biogenesis is usually accompanied by a reduced number of mitochondria or mitochondrial mass. Indeed, in the older diseased women, the mitochondrial mass analyzed by the mitochondrial DNA/nuclear DNA ratio was significantly reduced in comparison with the control. Surprisingly, the mitochondrial mass was similarly reduced in older male patients, even though no alterations in mitochondrial protein expression were detected in this patient group. We hypothesized that, in older male patients, this mitochondrial mass reduction may be due to the enhanced mitochondrial clearance via autophagy, which may be caused by elevated AMPK activity (Wang et al., 2018). Indeed, AMPK may promote the expression and activation of autophagy proteins (Asakura & Kitakaze, 2009). Expression analysis of autophagy markers in the present study revealed an upregulation of at least two autophagy genes, that is, ATG5 and SQSTM1, in older men with DCMI, suggesting enhanced autophagy (Kuma et al., 2004; Pyo et al., 2013; Zhang et al., 2019). It is worth noting that these autophagy markers ATG5 and SQSTM1 were also upregulated in older female patients, but only at the protein level. Though the interpretation of these data is complex, based on previous animal studies (Triolo et al., 2022), one may posit that the accumulation of autophagy markers at the protein level in older female patients is due to disturbed autophagy.

In agreement with our results, alterations in mitochondrial biogenesis have been reported in various forms of cardiomyopathy (Flarsheim et al., 1996; Rosca & Hoppel, 2013) however, the sex and age differences have been the subject of little investigation. Our recent report (Barcena et al., 2020) characterized impaired mitochondrial biogenesis in older patients with dilated end‐stage cardiomyopathy, which was sex‐independent. Therefore, age‐ and sex‐dependent impairment of mitochondrial biogenesis may vary among different cardiomyopathy forms.

Posttranslational modification is a key regulator of mitochondrial enzymes' activity, and therefore mitochondrial function. Acetylation of mitochondrial proteins is increased in a failing heart (Horton et al., 2016; Parodi‐Rullan et al., 2018), which may lead to the decreased activity of various mitochondrial enzymes, such as SOD2, succinate dehydrogenase, pyruvate dehydrogenase, and ATP synthase (Horton et al., 2016; Zhang et al., 2018). To explore the acetylation state in mitochondrial matrix proteins, we applied a widely used marker, that is, SOD2 acetylation. Though no effect of cardiomyopathy was found in the female patients, significant and age‐dependent alterations of SOD2 acetylation were found in male patients: it was elevated in younger and reduced in older men. This reduced SOD2 acetylation appears to contradict previous reports proposing the existence of mitochondrial protein hyperacetylation in failing hearts (Parodi‐Rullan et al., 2018). One reason for the contradiction may be the preserved Sirt3 expression seen in our study, as other studies have reported a strong Sirt3 downregulation in end‐stage cardiomyopathy (Song et al., 2021; Sundaresan et al., 2015). Furthermore, the differences in acetyltransferase activity or NAD+ availability may be responsible for the contradiction. Thus, older male patients with DCMI may be the only group to benefit from the enhanced deacetylation of mitochondrial proteins and, in particular, SOD2, which is a key antioxidative mitochondrial enzyme.

In addition to mitochondrial dysfunction, heart failure is also associated with an increased pro‐inflammatory response (Hoffmann et al., 2019; Yue & Yao, 2016), and there are interactions between mitochondrial dysfunction and inflammation in hearts. For example, pro‐inflammatory mediators including IL‐1β, IL‐6, and TNF‐α promote a reduction in the NAD+/NADH ratio, leading to an impairment in mitochondrial biogenesis (Hahn et al., 2014), while mitochondrial dysfunction may induce cardiac inflammation via the release of mitochondrial DNA or formation of ROS (Liao et al., 2013; Oka et al., 2012).

In keeping with the beneficial mitochondrial biology in older male patients observed in this study, the expression of the master regulators of the inflammatory response, that is, NF‐κB and TLR4 expression, was significantly reduced in this group. When further analyzing the expression of various pro‐inflammatory cytokines—IL‐1β (data not shown), IL‐12, and IL‐18—we found significant upregulation only in IL‐18 mRNA expression in older women with DCMI. The alteration in the IL‐18 expression seems to be age‐dependent as this factor was not affected in younger patients with DCMI. In agreement with this finding, several studies have also reported an IL‐18 increase in older individuals (Dinarello, 2009; Ferrucci et al., 2005; Franceschi et al., 2007). Importantly, TLR4 is a promoter of IL‐18 expression in cardiomyocytes (Liu et al., 2015) however, our results demonstrated a TLR4‐independent upregulation of the IL‐18 expression in the heart of older female patients.

Cardiomyopathy is accompanied by a decline in anti‐inflammatory cytokines (Barcena et al., 2022; Kaur et al., 2022). In one of our recent studies, we observed the sex‐independent downregulation of IL‐10 in older patients with end‐stage dilated cardiomyopathy (Barcena et al., 2020). In the present study, we also observed a marked downregulation of cardiac IL‐10 in younger male and female patients. However, in older patients, a significant IL‐10 downregulation was found only in men.

Several forms of cardiomyopathy are associated with cardiac senescence (Mehdizadeh et al., 2022). In the present study, changes in the expression of senescence markers, like loss of lamin B1, were observed only in elderly DCMI patients in a sex‐independent manner. It is well accepted that the increased expression of pro‐inflammatory cytokines and chemokines promotes the development of a senescence‐associated secretory phenotype (SASP) (Coppe et al., 2010; Furman et al., 2019). In accordance, in the present study expression analysis of several SASP molecules revealed elevation of VEGF, specifically in older female patients, suggesting the role of the pro‐inflammatory shift in this patient group.

Regarding fibrosis in cardiac remodeling, the chronic activation of the inflammatory markers promotes the upregulation of collagen deposition and pathological fibrosis formation in many diseases including myocarditis (Wynn, 2008). Several studies have shown that severe fibrosis formation is more common in male hearts with myocarditis (Asakura & Kitakaze, 2009; Barcena et al., 2021; Cavasin et al., 2006; Cocker et al., 2009; Coronado et al., 2012; Haddad et al., 2008) however, in our study, we also observed significant fibrosis formation in the hearts of older female patients with DCMI.

In conclusion, DCMI in older women is associated with the reduced expression of mitochondrial proteins and elevated IL‐18 and VEGF expression. These cardiomyopathy effects are absent in older male patients, which may be due to the significant elevation of AMPK expression and activity. In addition, mitochondrial homeostasis is further supported by reduced acetylation of mitochondrial proteins in older male patients.

## 5 LIMITATION OF THE STUDY

In this study, a small patient cohort was investigated, as the availability of human myocardial samples from both diseased and healthy individuals is limited. In addition, myocardial samples were obtained from apex tissue (patients with LVAD, 39%) or from the left lateral wall (patients who underwent heart transplantation, 61%). Nevertheless, the key effects observed in the study, for example, alterations in AMPK phosphorylation and expression of mitochondrial proteins, as well as SOD2 acetylation in older diseased versus healthy individuals, have sufficiently high robustness (at least twofold change) to ensure the validity of the conclusion.

One important limitation of the study is the medication of patients before samples have been obtained. Indeed, all patients were treated with numerous drugs, for example, ACE inhibitors, AT1 receptor blockers, beta‐blockers, or diuretics, which might have mitochondrial effects (for review see (Betiu et al., 2022)). Particularly, treatment with beta‐blockers may stimulate AMPK activity (Hu et al., 2019) and mitochondrial biogenesis (Yao et al., 2016). Similarly, AT1 receptor blockers and ACE inhibitors may upregulate Sirt1 and PGC‐1α, and promote mitochondrial biogenesis (Liu et al., 2021; Picca et al., 2018). Furthermore, beta‐blockers and ACE inhibitors might have immunoregulatory actions, leading to the modulation of several cytokines (Ohtsuka et al., 2001; Platten et al., 2009) Therefore, the results of the study should be taken with caution.

Another limitation of the study is the restriction of the statistical analyses to the comparison of DCMI versus controls. We avoided ANOVA analysis due to the small patient cohorts. Furthermore, the primary aim of the present study was to evaluate the effects of DCMI rather than the effects of sex or aging. Nevertheless, we also consider sex and age differences in the effects of DCMI. Furthermore, it would be interesting to analyze age as a continuous variable rather than categorical (young vs. old), to further confirm the age‐related differences.

In addition, interleukins in the present study were mostly analyzed at the mRNA level. This is a limitation, especially for interleukins synthesized as inactive pro‐interleukins, such as pro‐IL‐1β and pro‐IL‐18, which need to be cleaved enzymatically by caspase‐1 for activation.

Finally, humans are light‐sensitive organisms and are affected by circadian clocks that control daily changes in the expression of numerous genes throughout the body, including the heart (Durgan & Young, 2010). Because the human samples were obtained at different times, this limitation of the study should be considered.

## AUTHOR CONTRIBUTIONS

M.B. conceived the project, analyzed the data, prepared the figures, and wrote the main manuscript text. G.T. performed the real‐time PCR experiments, western blot experiments, IHC staining, and analyzed the data. N.H. performed the real‐time PCR, western blot experiments, and analyzed the data. P.B. performed the western blot experiments and analyzed the data. H.M. procured the human myocarditis tissue and revised the manuscript. I.B. procured the human tissue and revised the manuscript, U.M.W. revised the manuscript. Y.L. analyzed the data and wrote the main part of the manuscript, and V.R.Z. acquired research funds and coordinated the project. All authors commented on the manuscript.

## ACKNOWLEDGEMENTS

We thank Ms Summer Banks for the revision of the manuscript.

## FUNDING INFORMATION

This work was supported by the DZHK (German Centre for Cardiovascular Research), by the BMBF (German Ministry of Education and Research), and by the Ministry of Science, Research and Culture of the State of Brandenburg. Non‐diseased cardiac tissue collection and management were supported by the Hungarian National Research, Development, and Innovation Office (K‐128851 and TKP2021‐EGA‐32) to IB.

## CONFLICT OF INTEREST STATEMENT

The authors declare no competing interests.

## Supporting information


Data S1:

Figure S1:

## Data Availability

Data available on request from the authors.
