# Supplementary figures and images for: Sex and age differences in AMPK phosphorylation, mitochondrial homeostasis, and inflammation in hearts from inflammatory cardiomyopathy patients
Source: Aging Cell. 2023 Jun 26;22(8):e13894. doi: 10.1111/acel.13894 (PMC10410062; doi:10.1111/acel.13894)

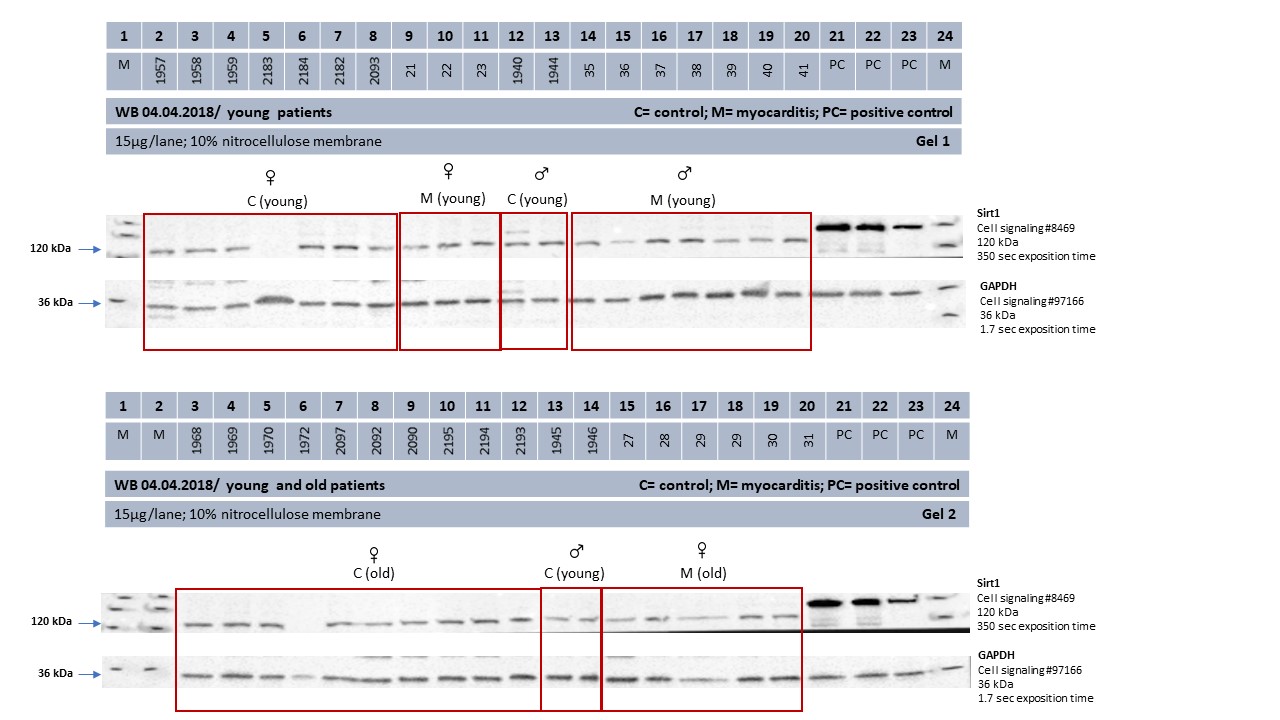

Supplement: Supplementary file 1 — Data S1: [file ACEL-22-e13894-s002.zip › Original blot 1.JPG]

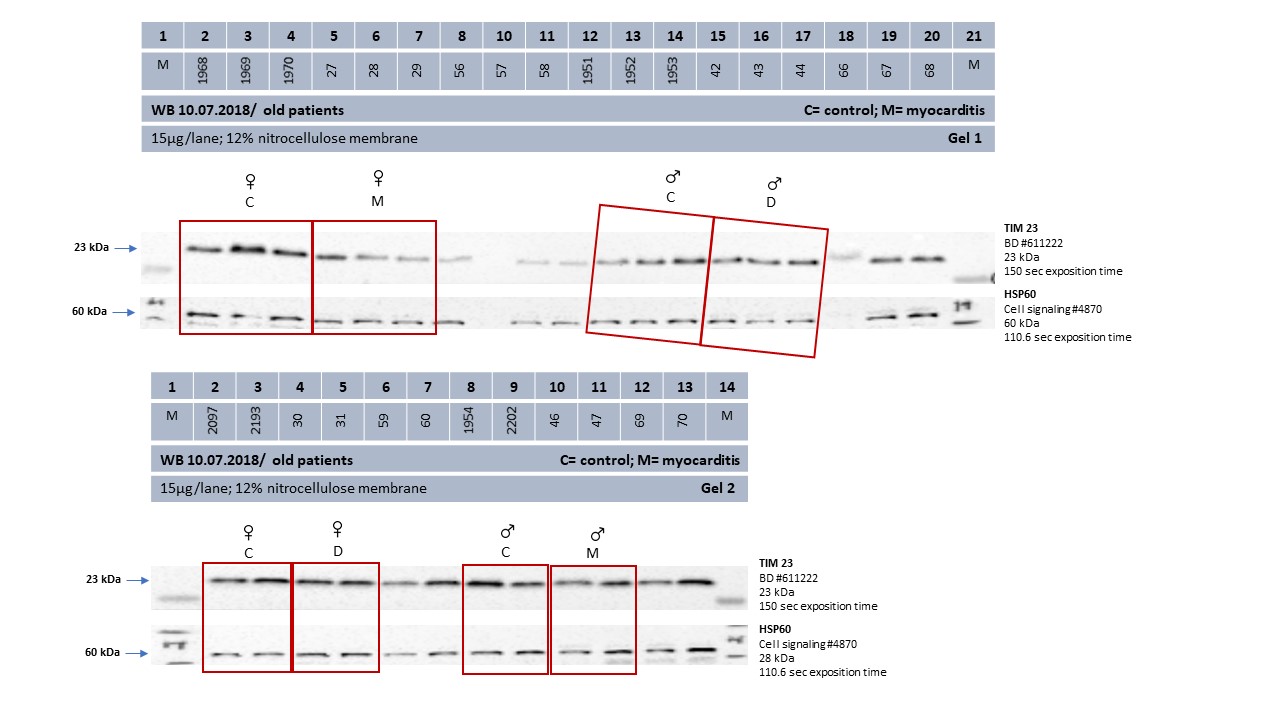

Supplement: Supplementary file 1 — Data S1: [file ACEL-22-e13894-s002.zip › Original blot 10.JPG]

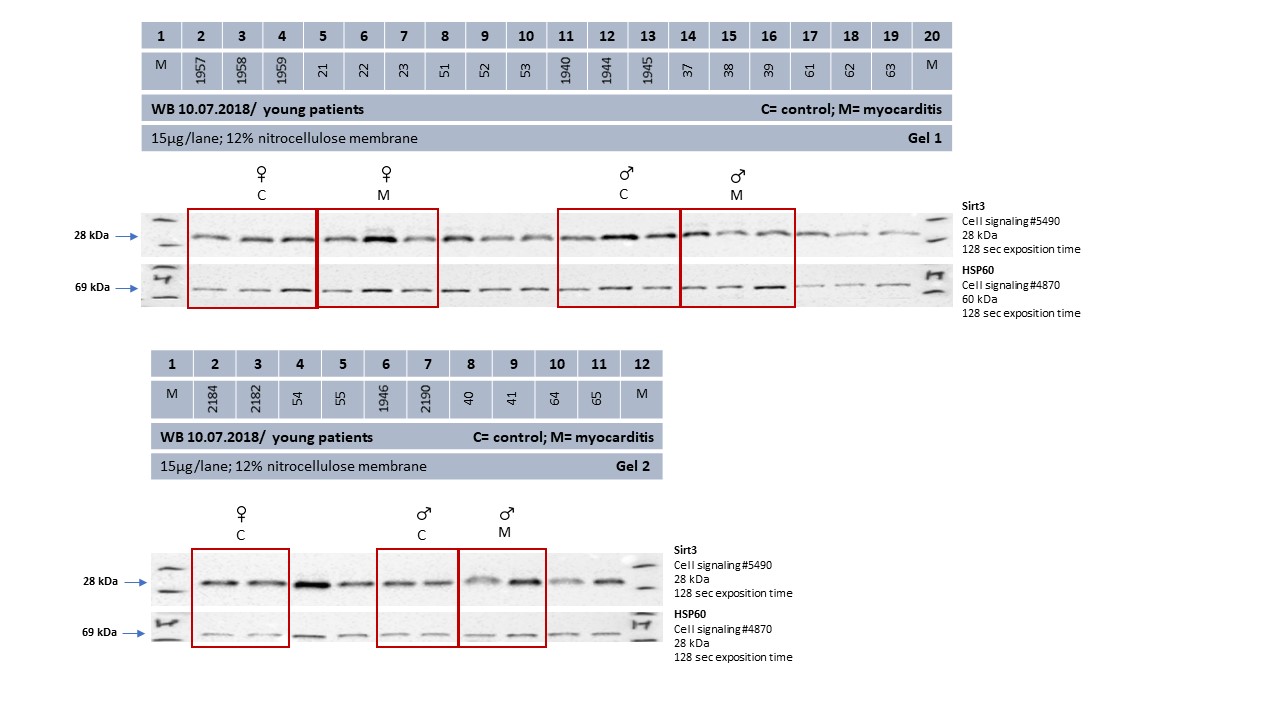

Supplement: Supplementary file 1 — Data S1: [file ACEL-22-e13894-s002.zip › Original blot 11.JPG]

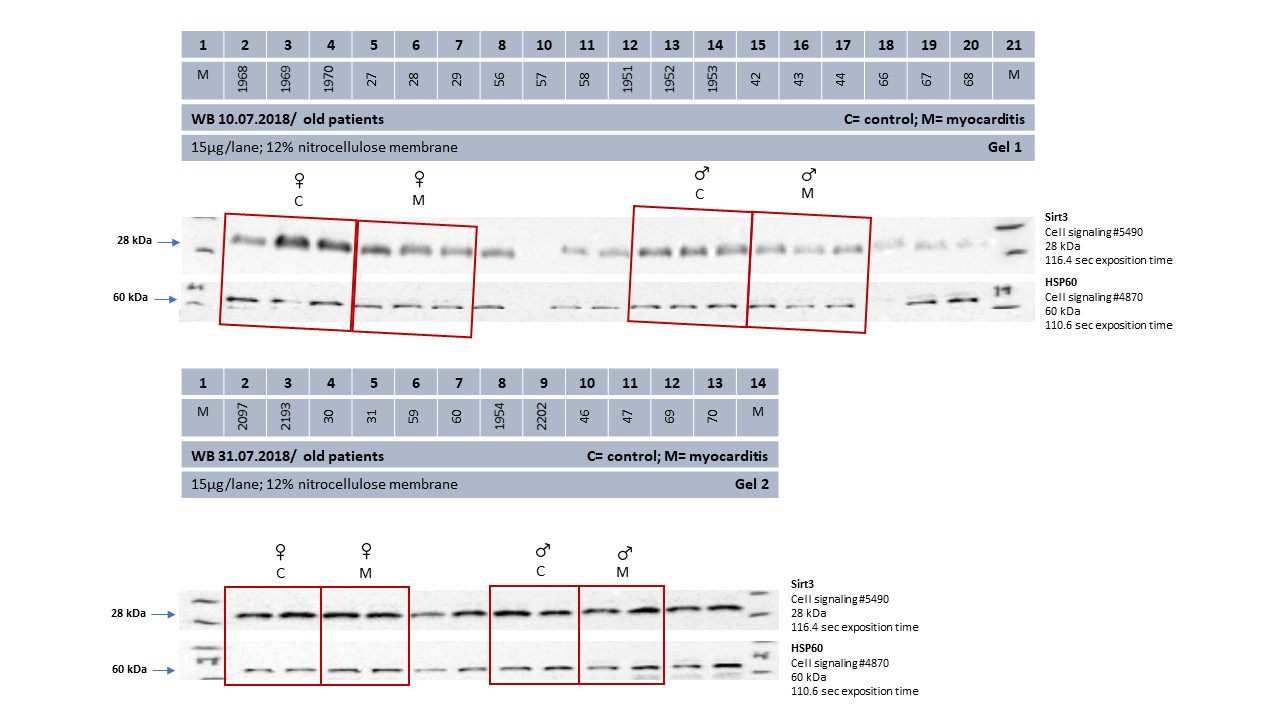

Supplement: Supplementary file 1 — Data S1: [file ACEL-22-e13894-s002.zip › Original blot 12.JPG]

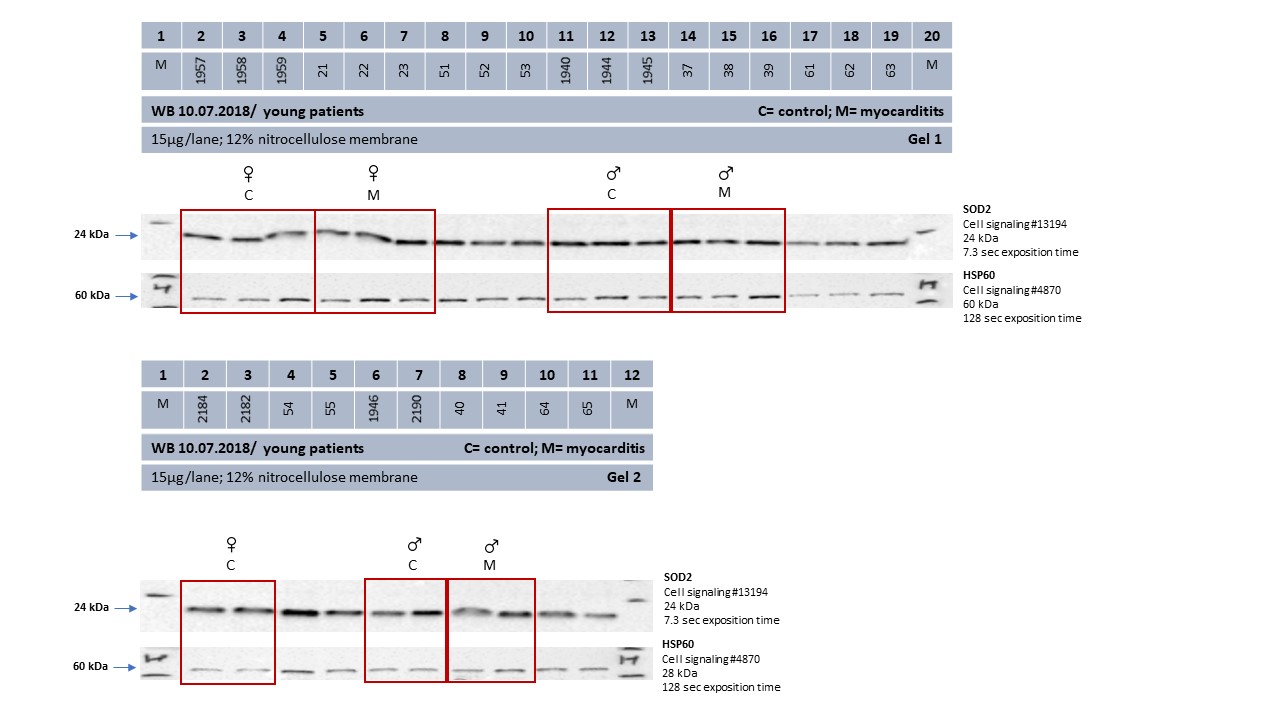

Supplement: Supplementary file 1 — Data S1: [file ACEL-22-e13894-s002.zip › Original blot 13.JPG]

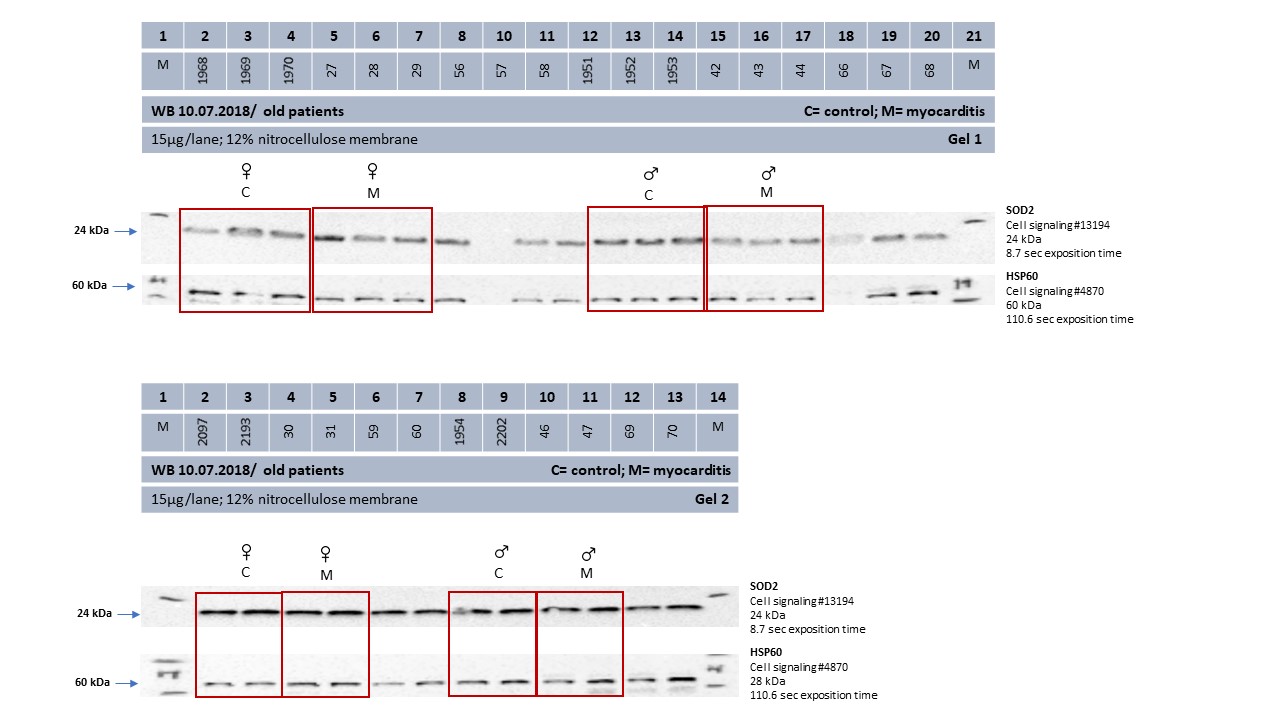

Supplement: Supplementary file 1 — Data S1: [file ACEL-22-e13894-s002.zip › Original blot 14.JPG]

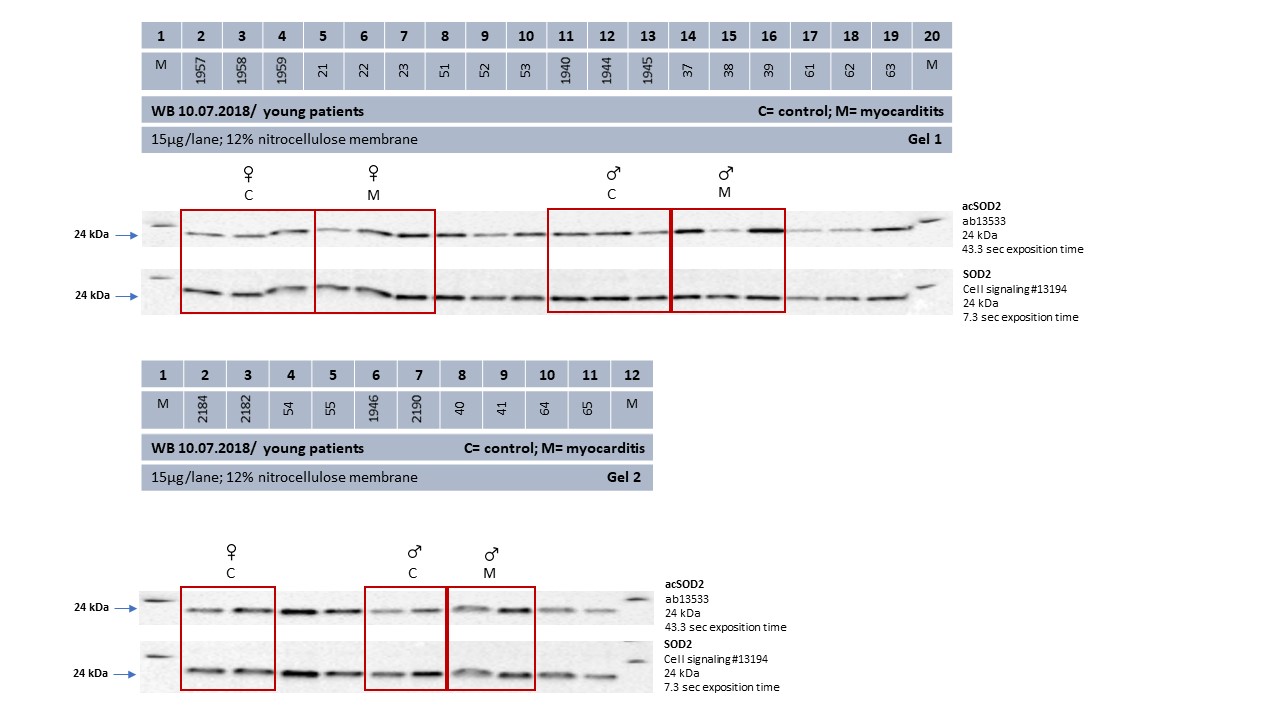

Supplement: Supplementary file 1 — Data S1: [file ACEL-22-e13894-s002.zip › Original blot 15.JPG]

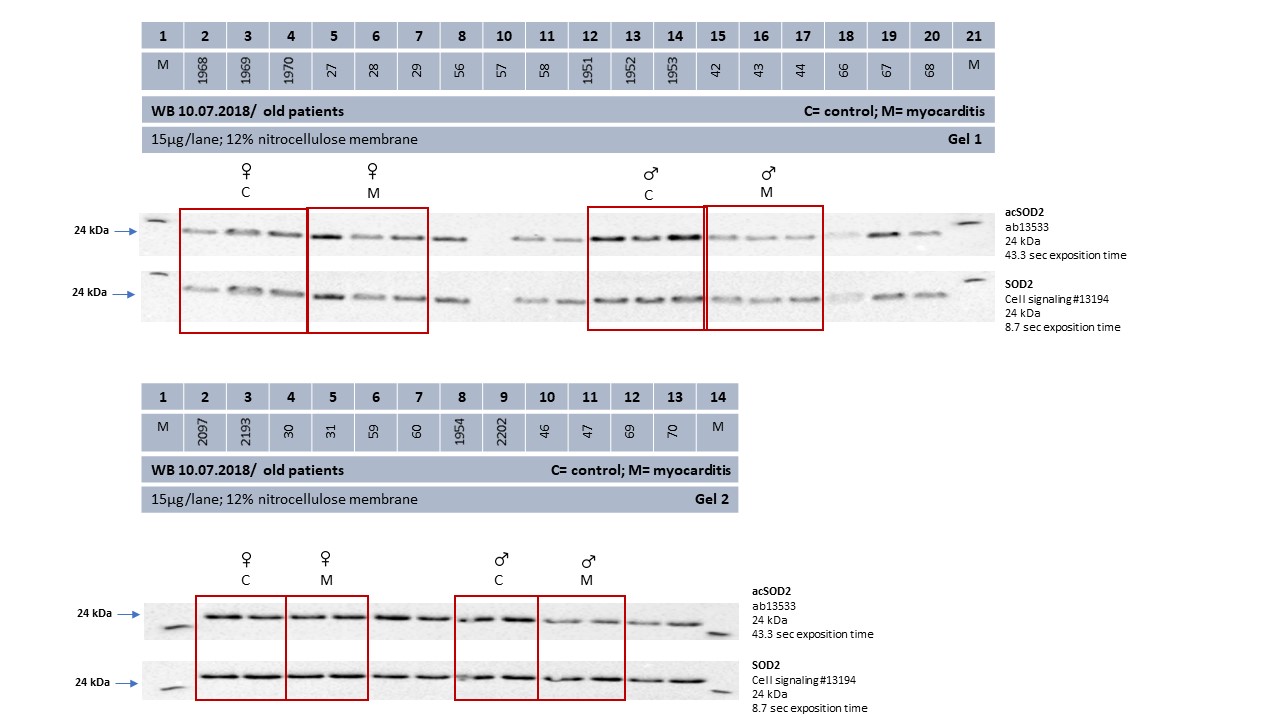

Supplement: Supplementary file 1 — Data S1: [file ACEL-22-e13894-s002.zip › Original blot 16.JPG]

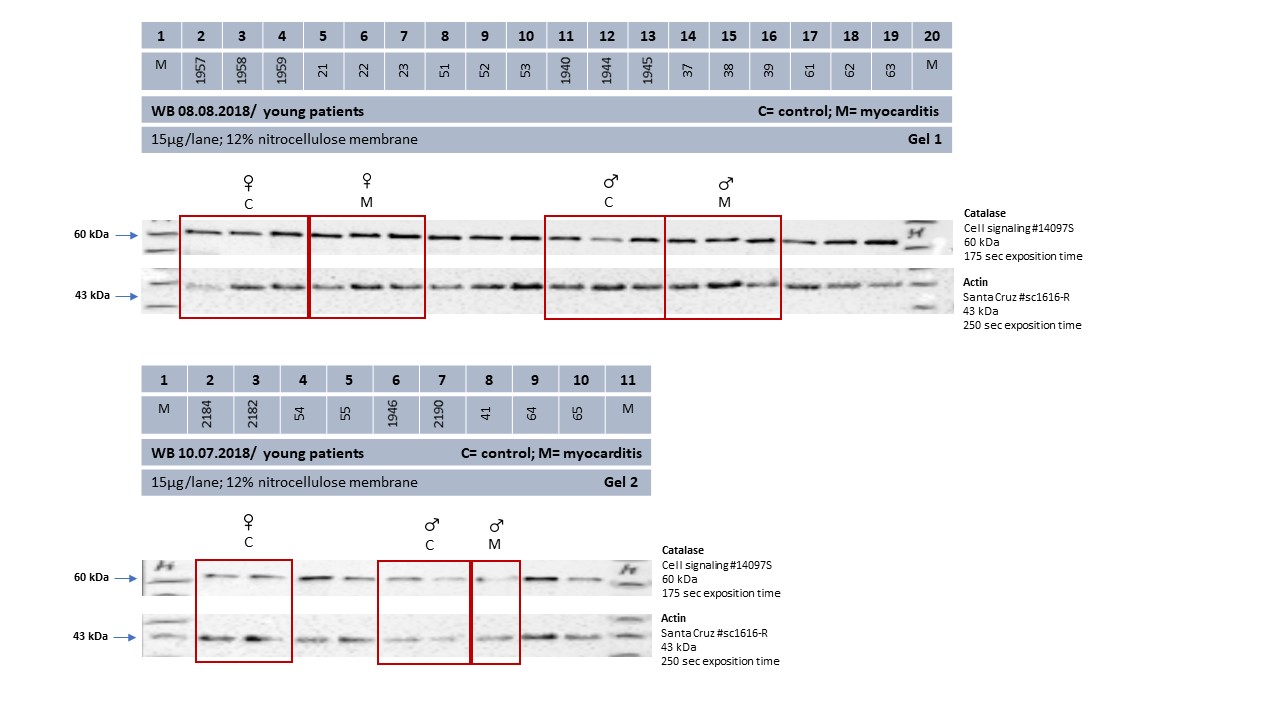

Supplement: Supplementary file 1 — Data S1: [file ACEL-22-e13894-s002.zip › Original blot 17.JPG]

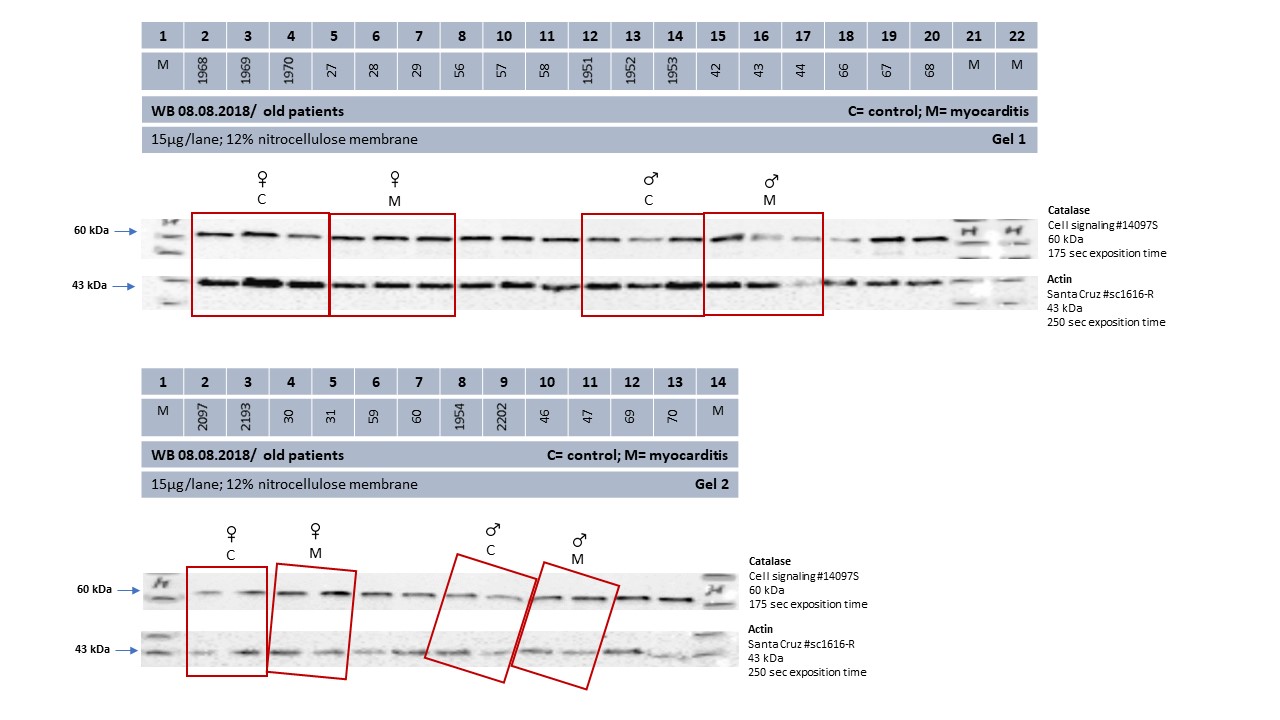

Supplement: Supplementary file 1 — Data S1: [file ACEL-22-e13894-s002.zip › Original blot 18.JPG]

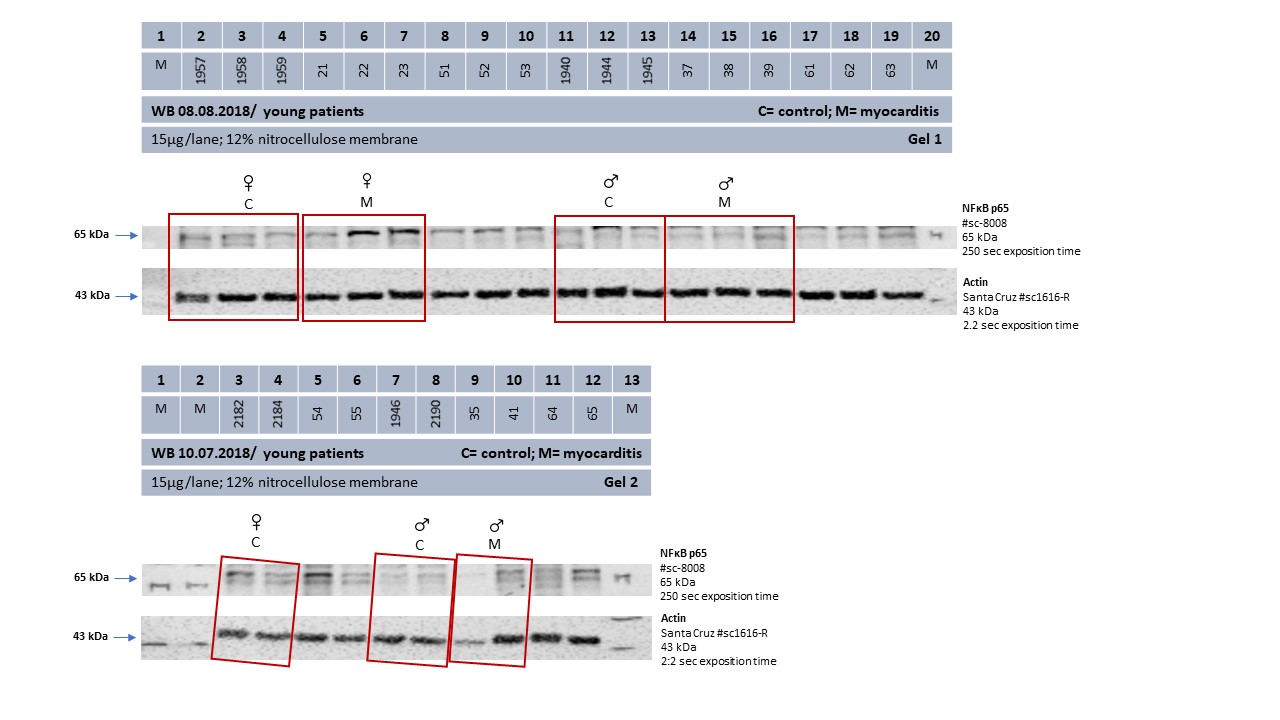

Supplement: Supplementary file 1 — Data S1: [file ACEL-22-e13894-s002.zip › Original blot 19.JPG]

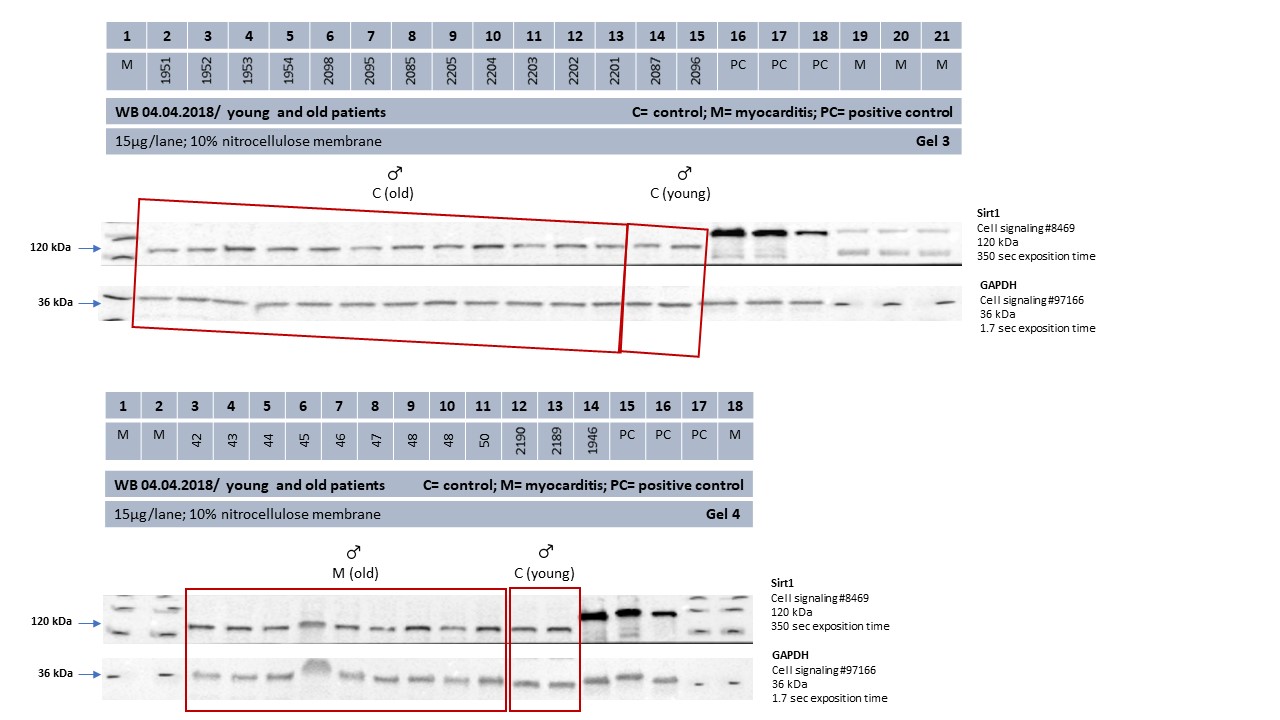

Supplement: Supplementary file 1 — Data S1: [file ACEL-22-e13894-s002.zip › Original blot 2.JPG]

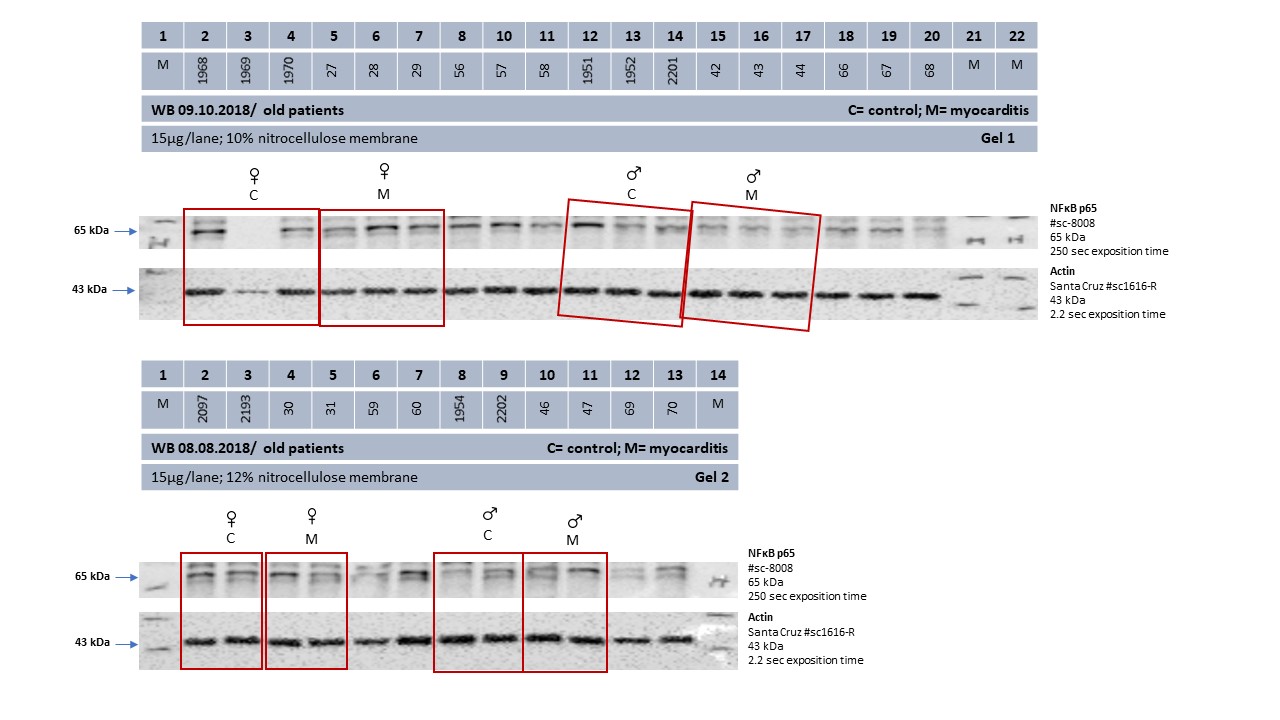

Supplement: Supplementary file 1 — Data S1: [file ACEL-22-e13894-s002.zip › Original blot 20.JPG]

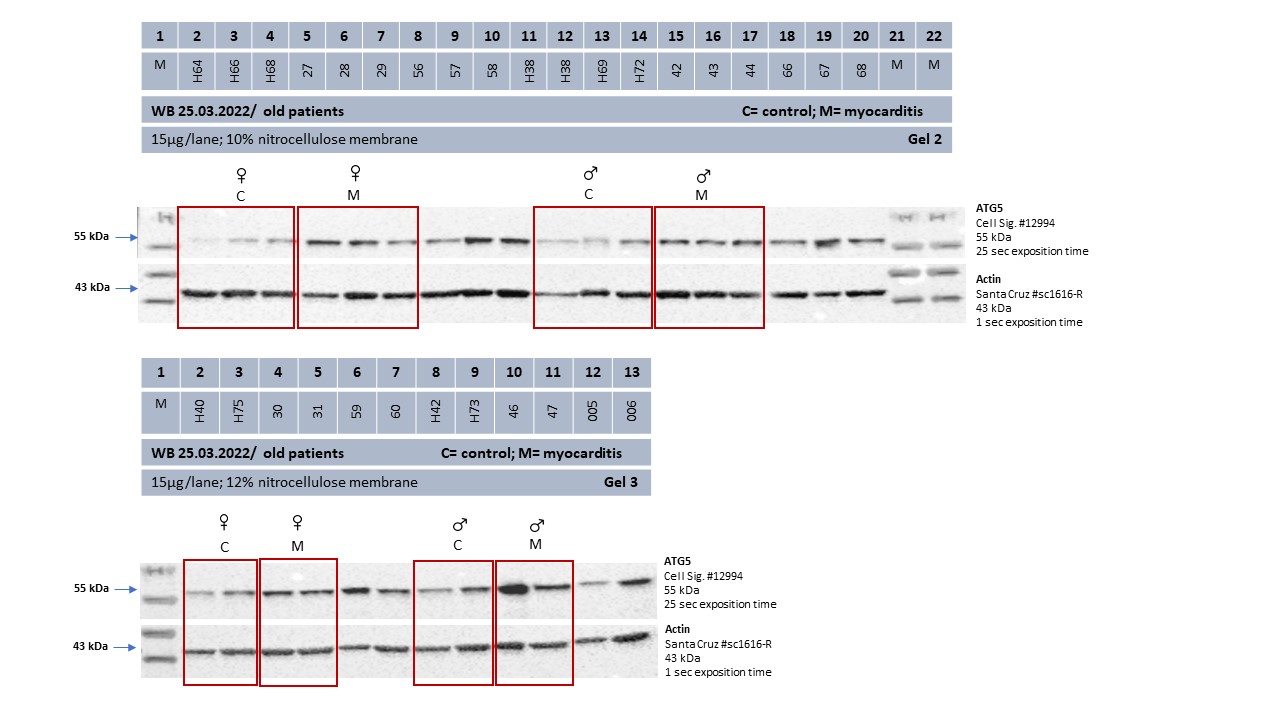

Supplement: Supplementary file 1 — Data S1: [file ACEL-22-e13894-s002.zip › Original blot 21.JPG]

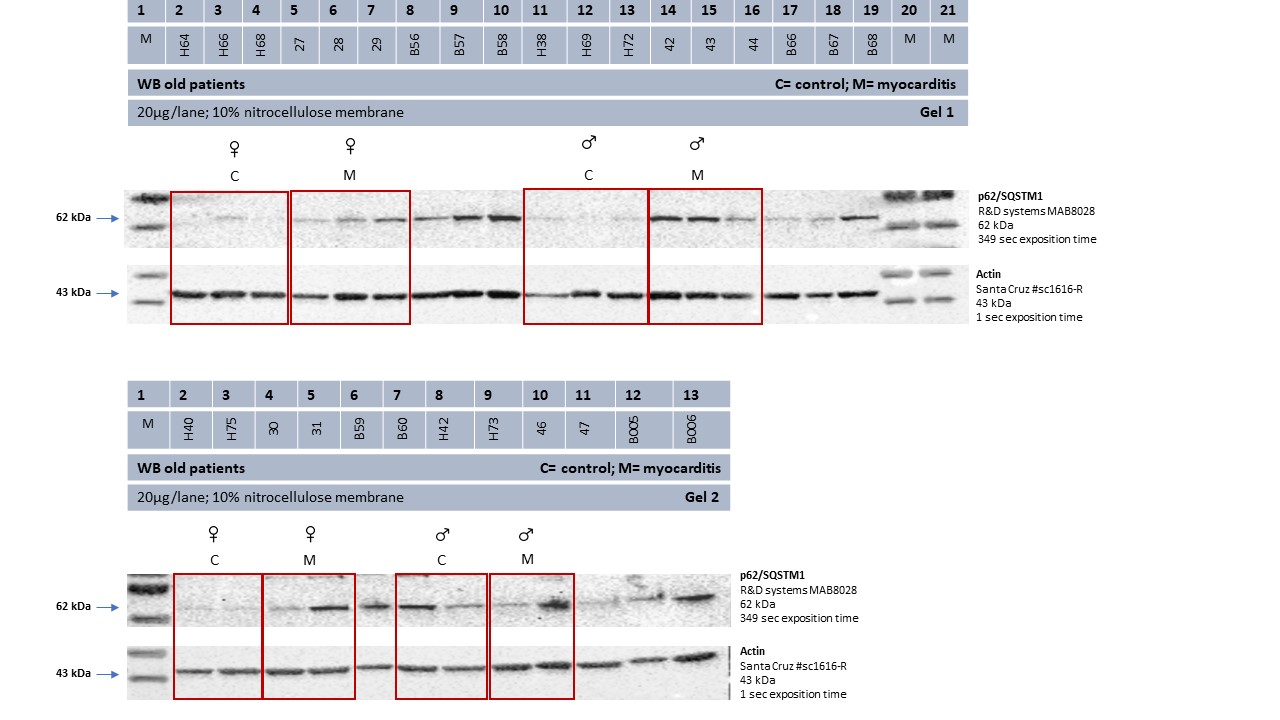

Supplement: Supplementary file 1 — Data S1: [file ACEL-22-e13894-s002.zip › Original blot 22.JPG]

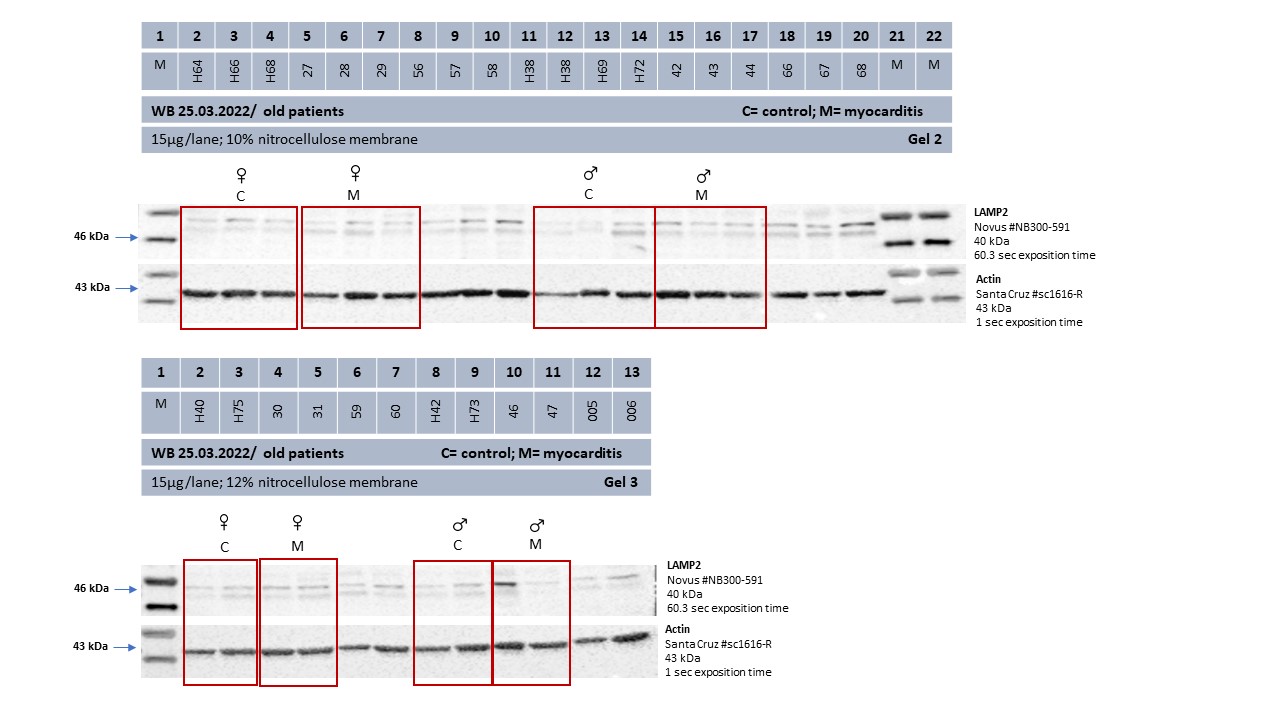

Supplement: Supplementary file 1 — Data S1: [file ACEL-22-e13894-s002.zip › Original blot 23.JPG]

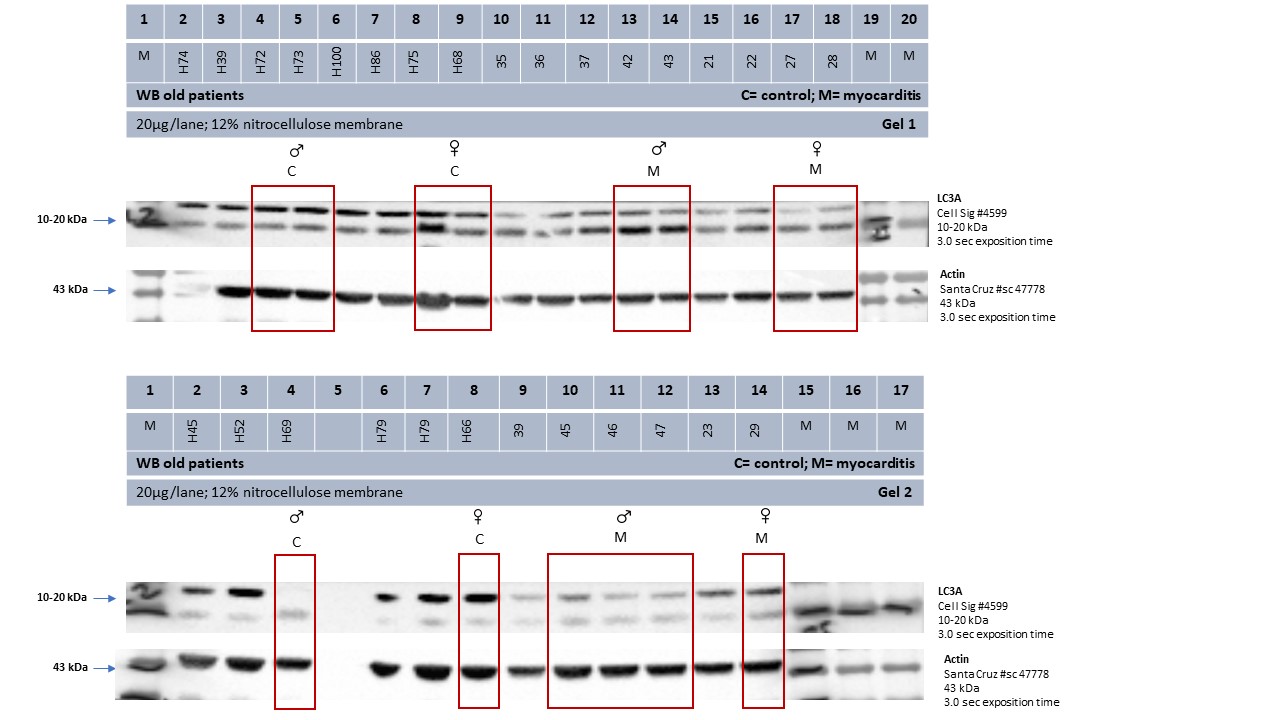

Supplement: Supplementary file 1 — Data S1: [file ACEL-22-e13894-s002.zip › Original blot 24.JPG]

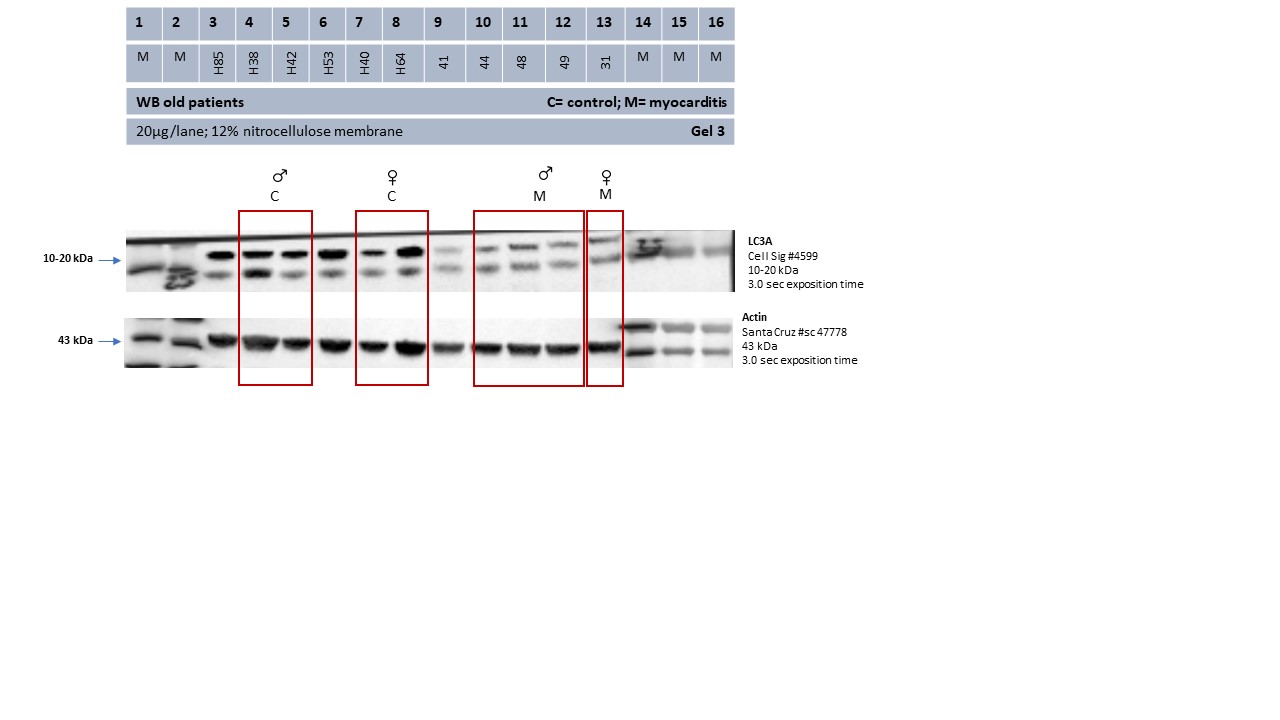

Supplement: Supplementary file 1 — Data S1: [file ACEL-22-e13894-s002.zip › Original blot 25.JPG]

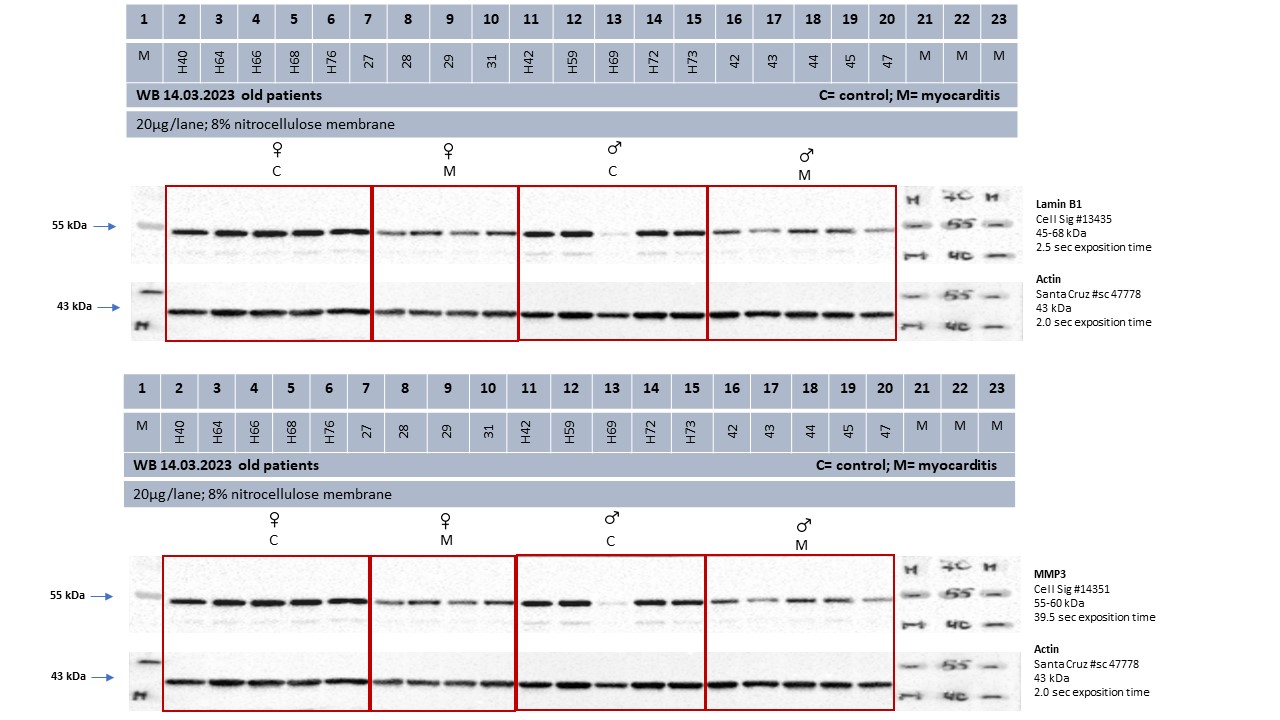

Supplement: Supplementary file 1 — Data S1: [file ACEL-22-e13894-s002.zip › Original blot 26.JPG]

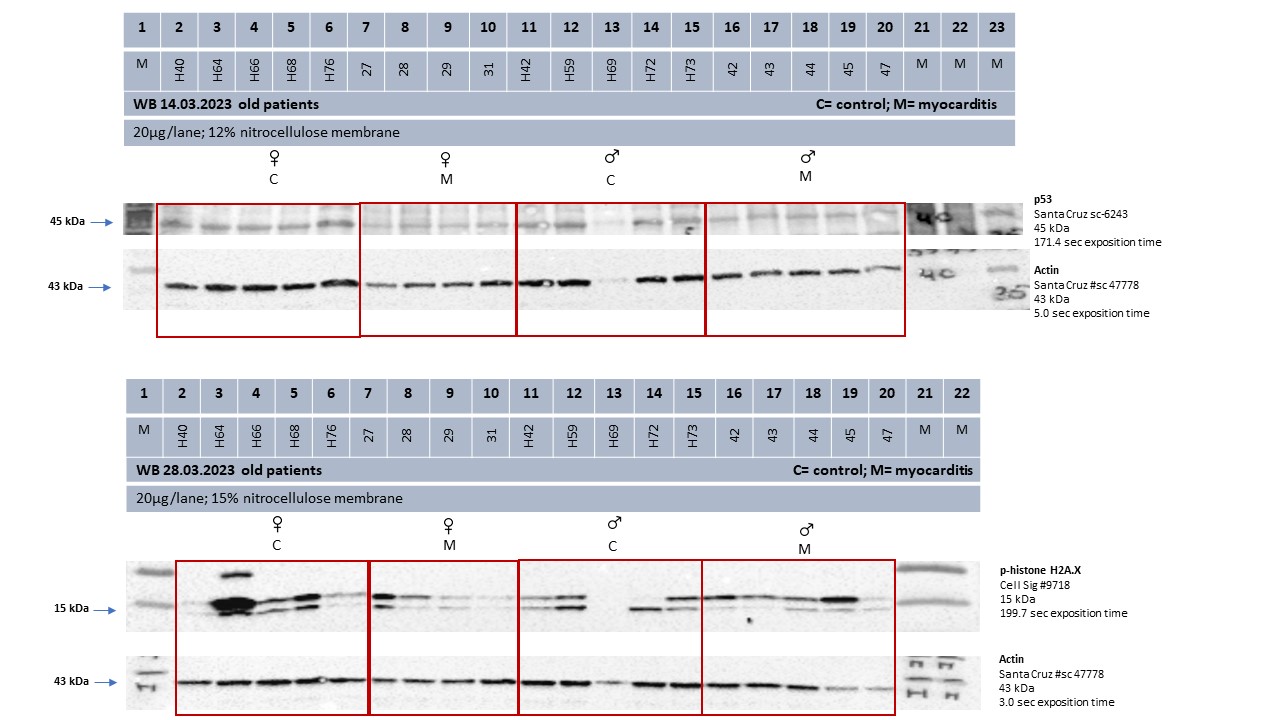

Supplement: Supplementary file 1 — Data S1: [file ACEL-22-e13894-s002.zip › Original blot 27.JPG]

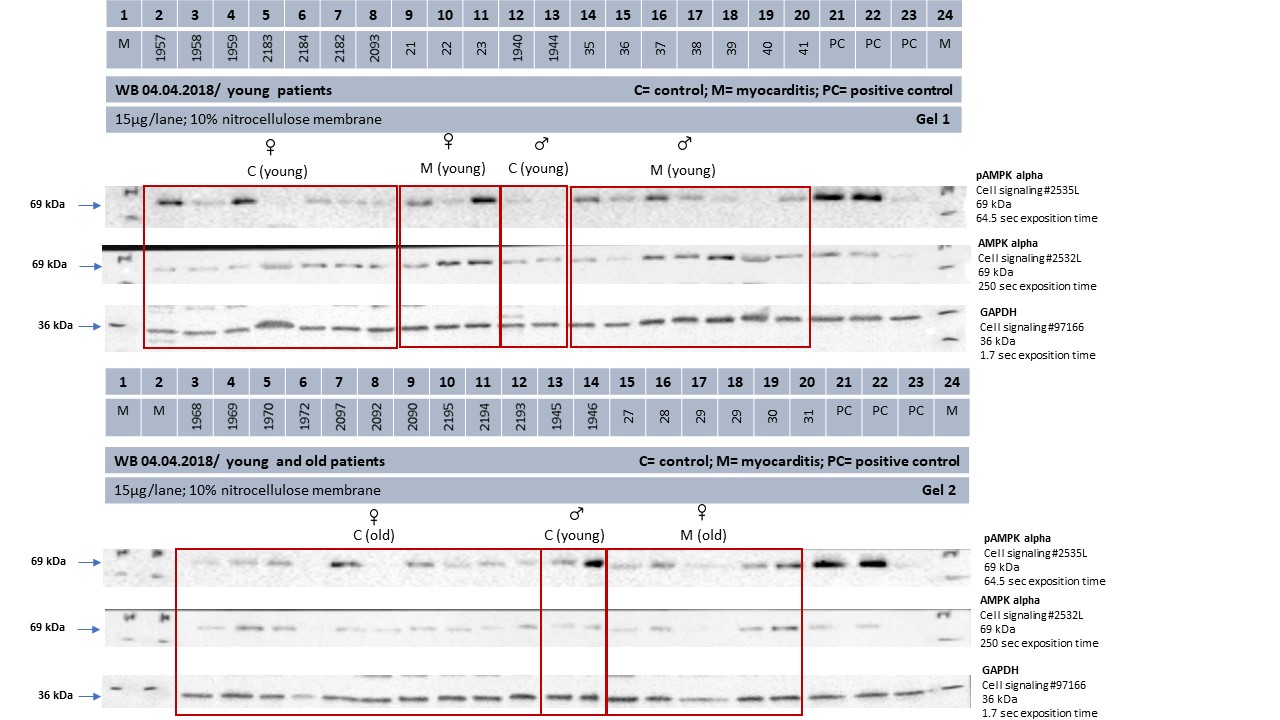

Supplement: Supplementary file 1 — Data S1: [file ACEL-22-e13894-s002.zip › Original blot 3.JPG]

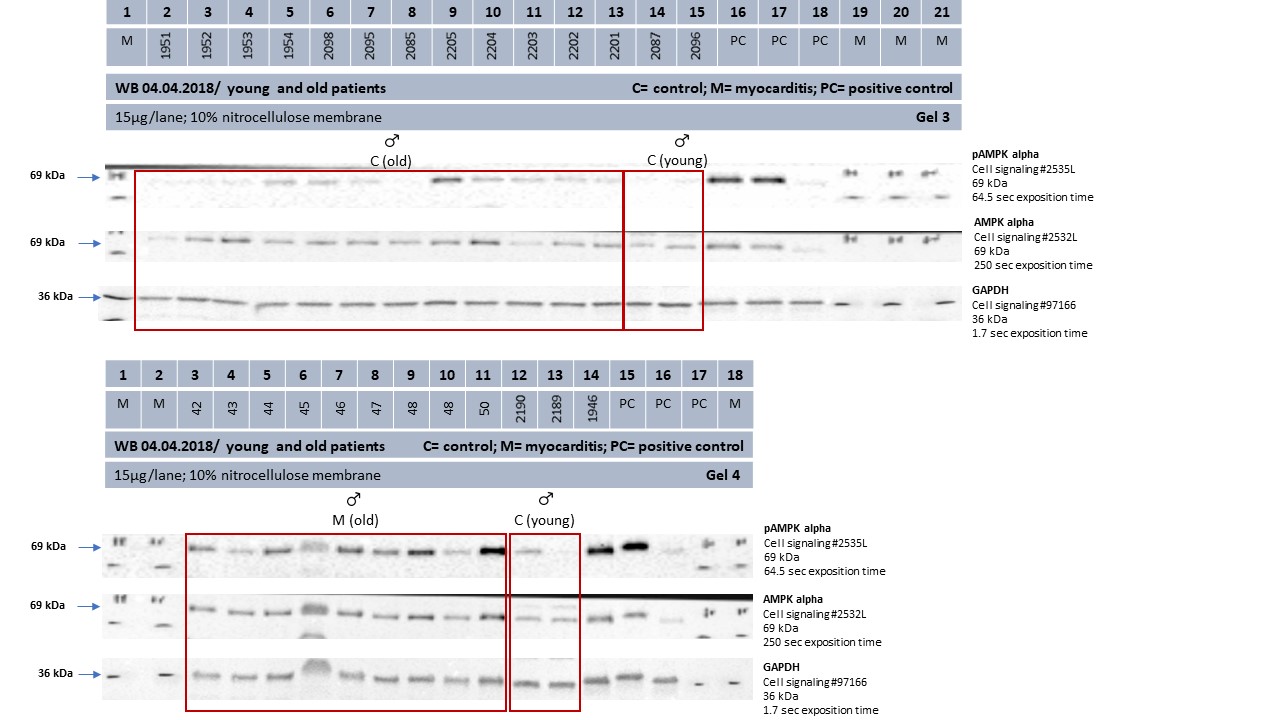

Supplement: Supplementary file 1 — Data S1: [file ACEL-22-e13894-s002.zip › Original blot 4.JPG]

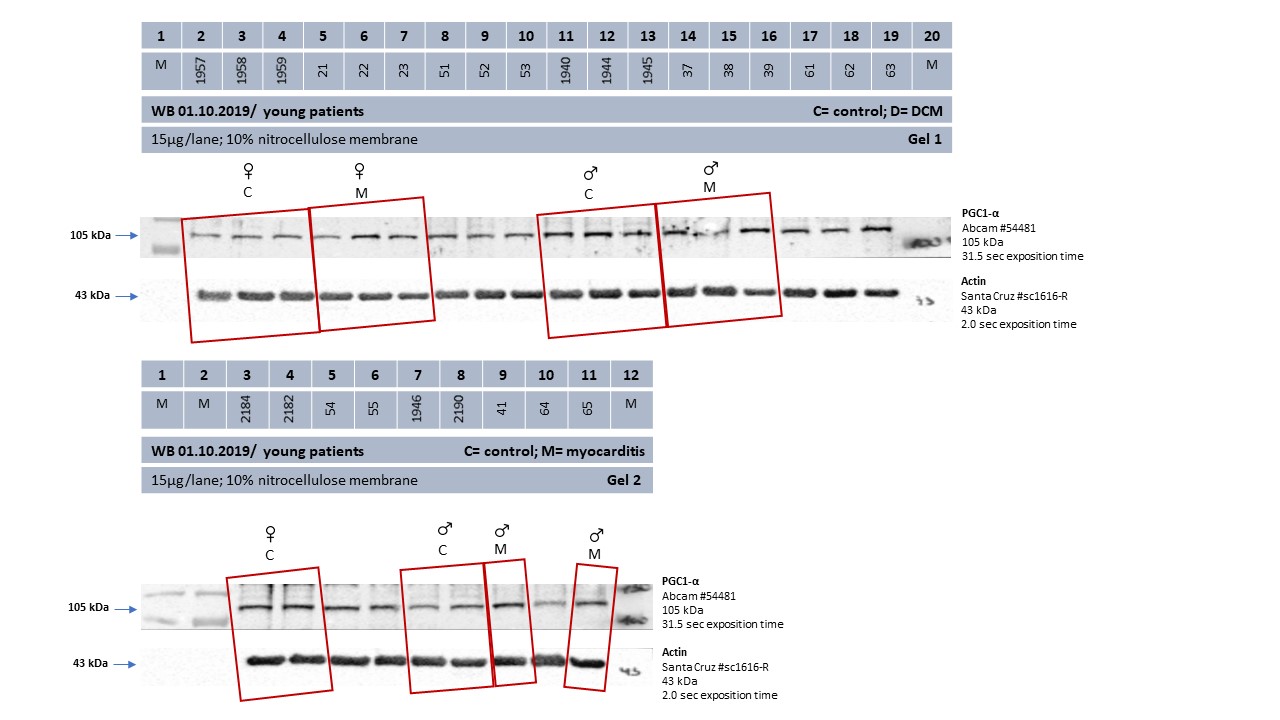

Supplement: Supplementary file 1 — Data S1: [file ACEL-22-e13894-s002.zip › Original blot 5.JPG]

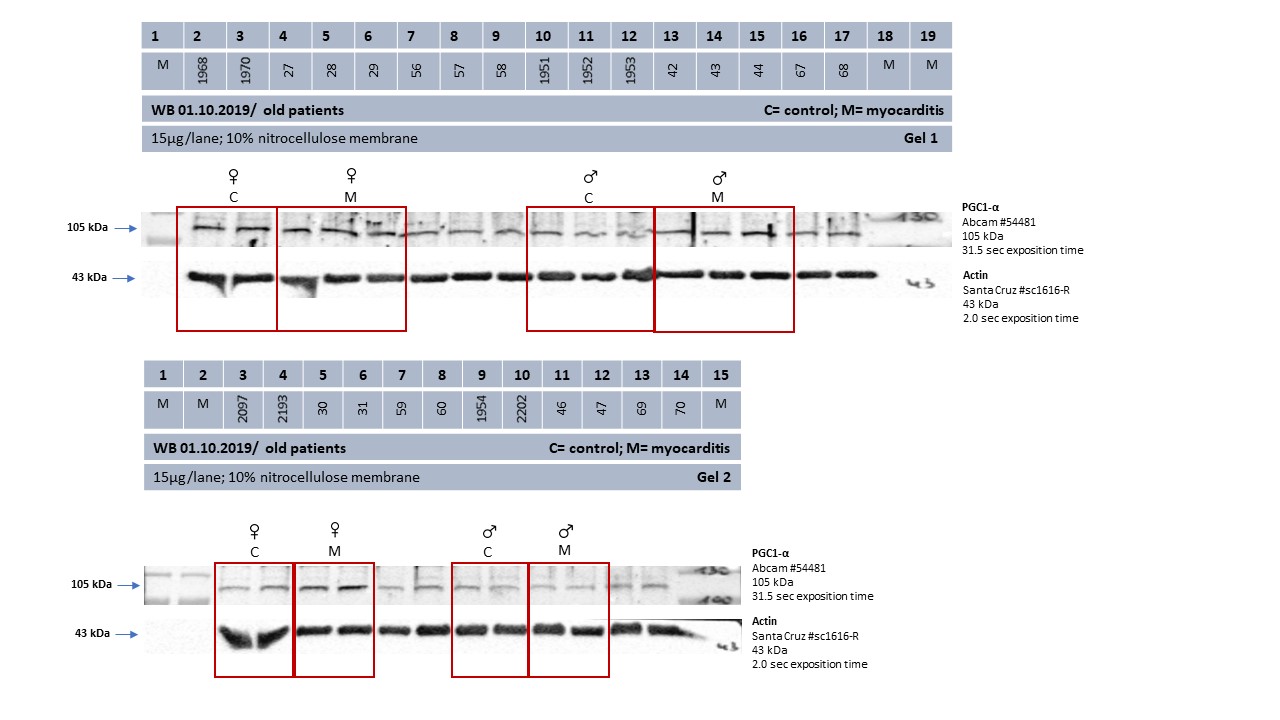

Supplement: Supplementary file 1 — Data S1: [file ACEL-22-e13894-s002.zip › Original blot 6.JPG]

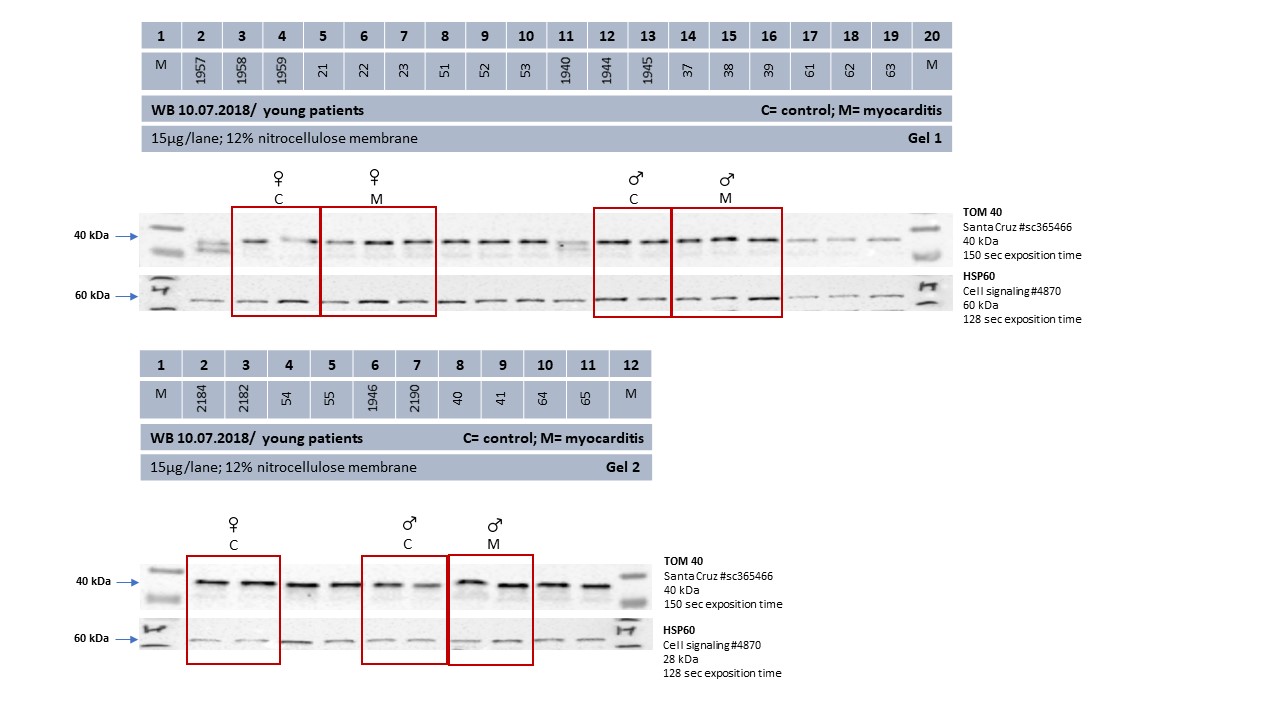

Supplement: Supplementary file 1 — Data S1: [file ACEL-22-e13894-s002.zip › Original blot 7.JPG]

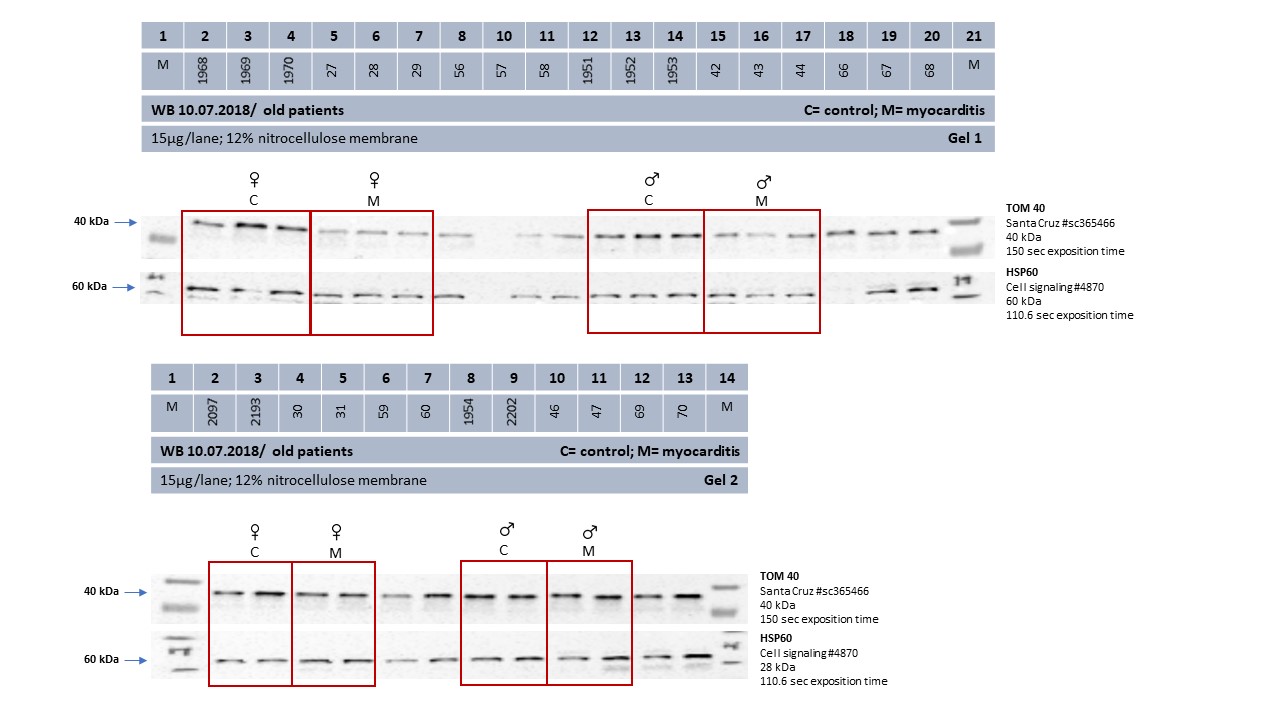

Supplement: Supplementary file 1 — Data S1: [file ACEL-22-e13894-s002.zip › Original blot 8.JPG]

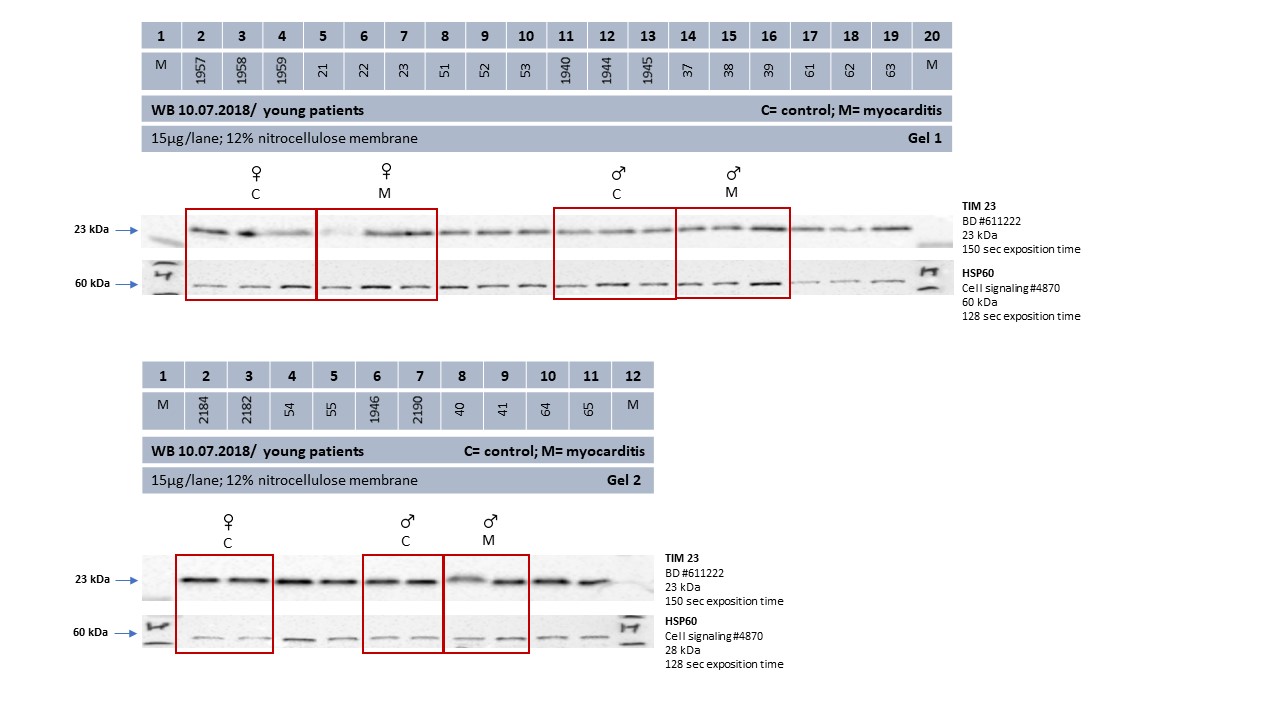

Supplement: Supplementary file 1 — Data S1: [file ACEL-22-e13894-s002.zip › Original blot 9.JPG]

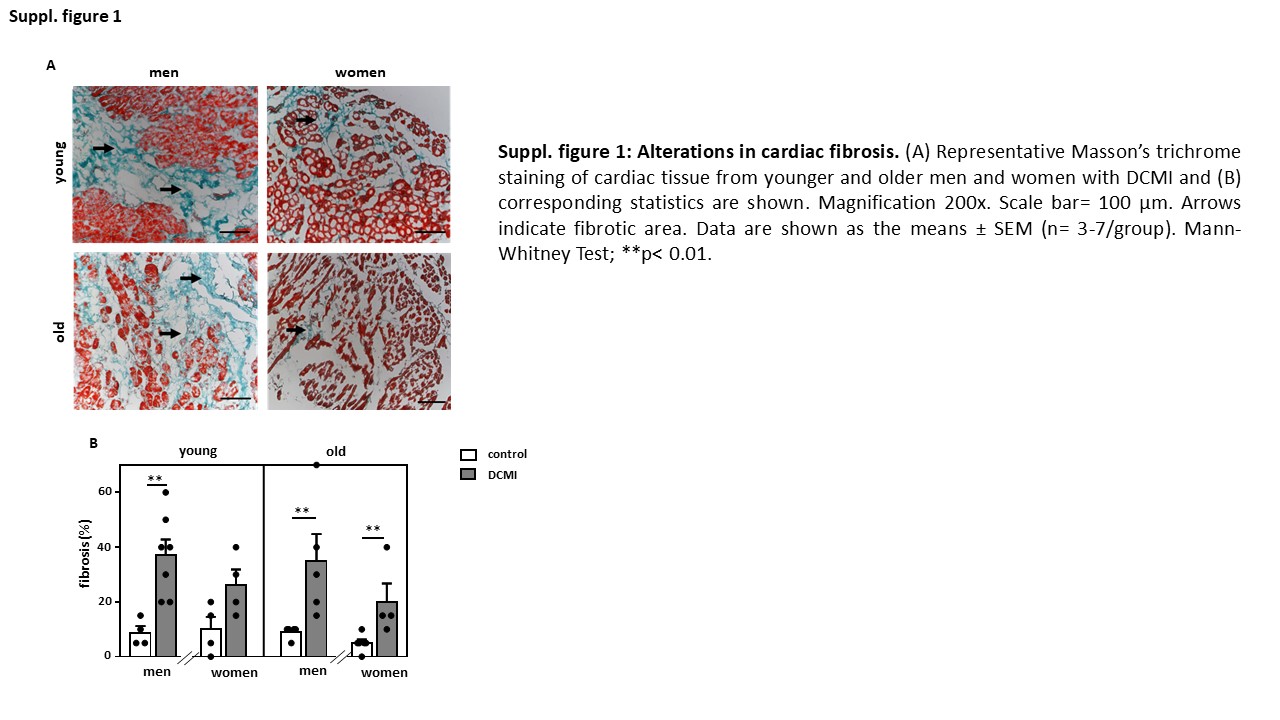

Supplement: Supplementary file 2 — Figure S1: [file ACEL-22-e13894-s001.jpg]
